# Borneol Ester Derivatives as Entry Inhibitors of a Wide Spectrum of SARS-CoV-2 Viruses

**DOI:** 10.3390/v14061295

**Published:** 2022-06-14

**Authors:** Olga I. Yarovaya, Dmitriy N. Shcherbakov, Sophia S. Borisevich, Anastasiya S. Sokolova, Maxim A. Gureev, Edward M. Khamitov, Nadezda B. Rudometova, Anastasiya V. Zybkina, Ekaterina D. Mordvinova, Anna V. Zaykovskaya, Artem D. Rogachev, Oleg V. Pyankov, Rinat A. Maksyutov, Nariman F. Salakhutdinov

**Affiliations:** 1 Department of Medicinal Chemistry, N.N. Vorozhtsov Novosibirsk Institute of Organic Chemistry SB RAS, Lavrentiev ave., 9, 630090 Novosibirsk, Russia; asokolova@nioch.nsc.ru (A.S.S.); mordvinova97@mail.ru (E.D.M.); artrogachev@yandex.ru (A.D.R.); anvar@nioch.nsc.ru (N.F.S.); 2 State Research Center of Virology and Biotechnology VECTOR, Rospotrebnadzor, 630559 Koltsovo, Russia; dnshcherbakov@gmail.com (D.N.S.); andreeva_nb@vector.nsc.ru (N.B.R.); zybkina_av@vector.nsc.ru (A.V.Z.); zaykovskaya_av@vector.nsc.ru (A.V.Z.); pyankov_ov@vector.nsc.ru (O.V.P.); maksyutov_ra@vector.nsc.ru (R.A.M.); 3 Laboratory of Chemical Physics Ufa Institute of Chemistry, Ufa Federal Research Center, RAS, Octyabrya pr., 71, 450054 Ufa, Russia; monrel@mail.ru (S.S.B.); khamitovem@gmail.com (E.M.K.); 4 Research Center “Digital Biodesign and Personalized Healthcare”, I.M. Sechenov First Moscow State Medical University, Trubetskaya str., 8/2, 119991 Moscow, Russia; max_technik@mail.ru; 5 Department of Computational Biology, Sirius University of Science and Technology, Olympic Ave., 1, 354340 Sochi, Russia

**Keywords:** SARS-CoV-2, coronavirus surface protein S-spike, pseudoviral system, terpene, borneol, molecular dynamics, molecular docking

## Abstract

In the present work we studied the antiviral activity of the home library of monoterpenoid derivatives using the pseudoviral systems of our development, which have glycoproteins of the SARS-CoV-2 virus strains Wuhan and Delta on their surface. We found that borneol derivatives with a tertiary nitrogen atom can exhibit activity at the early stages of viral replication. In order to search for potential binding sites of ligands with glycoprotein, we carried out additional biological tests to study the inhibition of the re-receptor-binding domain of protein S. For the compounds that showed activity on the pseudoviral system, a study using three strains of the infectious SARS-CoV-2 virus was carried out. As a result, two leader compounds were found that showed activity on the Wuhan, Delta, and Omicron strains. Based on the biological results, we searched for the potential binding site of the leader compounds using molecular dynamics and molecular docking methods. We suggested that the compounds can bind in conserved regions of the central helices and/or heptad repeats of glycoprotein S of SARS-CoV-2 viruses.

## 1. Introduction

The first reported cases of SARS-CoV-2-induced pneumonia came from China in late 2019 [1]. COVID-19 expanded from a localized outbreak to a pandemic in a matter of days. As of 10 April 2022, the total number of confirmed infections worldwide had reached 496 million, and the death toll had exceeded 6 million [2]. At the same time, the development of a number of vaccines against COVID-19 allowed immunization campaigns to be launched in the hope of achieving collective immunity by vaccinating the majority of the population [3].

The etiological agent of COVID-19 is the SARS-CoV-2 coronavirus. Coronaviruses are enveloped RNA-containing viruses with a genome of up to 30,000 base pairs in size and a virion diameter of approximately 120 nm [4]. A characteristic feature of viral particles is the S spikes on their surface. Coronaviruses pathogenic to humans include four viruses responsible for about a third of colds (HCoV 229E, OC43, NL63, and HKU1), and three viruses that have caused epidemics in the past two decades involving significant mortality: SARS-CoV-1 (2002–2003, ~10% mortality), MERS-CoV (Middle East respiratory syndrome coronavirus; 2012, ~35% mortality), and SARS-CoV-2 [5]. However, only the latest SARS-CoV-2 virus is extremely contagious, and this has already allowed it to be referred to as the most infectious agent of the century [6].

The main efforts of countries to control the COVID-19 pandemic have focused on the development and introduction of vaccines. Current vaccines against COVID-19 target the surface protein SARS-CoV-2, which contains the sites responsible for cell-to-cell interaction and penetration. It is the S protein that contains epitopes of neutralizing antibodies capable of protecting the organism [7,8]. At the same time, the vaccines have drawbacks. This is especially true for SARS-CoV-2, which is highly variable. On the one hand, a relatively rapid change in the epitope surface protein allows the virus to evade immunity developed after the use of a vaccine based on the previous variant of SARS-CoV-2 [9,10]. On the other hand, vaccines themselves, developed in great haste and often in violation of generally accepted testing deadlines, have side effects, many of which may not yet be understood at this time [11]. All of this necessitates the search for additional countermeasures against COVID-19. One approach is the use of specific antiviral therapy.

Using the conserved sites of key SARS-CoV-2 virus proteins as targets for low-molecular-weight antiviral drugs may be a more successful approach than a vaccine in both prevention and treatment [12]. One of the key interactions of a SARS-CoV-2 with a cell is the interaction between the receptor binding domain (RBD) of the S glycoprotein and Angiotensin-converting enzyme 2 (ACE2). Generally, a low molecular weight antiviral drug is easier to produce, store, deliver, and administer [13].

With the global workload of virology laboratories, methods for screening candidate substances using surrogate systems such as pseudoviruses [14,15], cellular systems [13], [16] and recombinant proteins are of particular importance [17,18,19]. Such systems avoid working with a live virus, increase safety and speed, and, most importantly, allow us to study the mechanisms and targets of action of potential antiviral substances.

**Figure 1:**
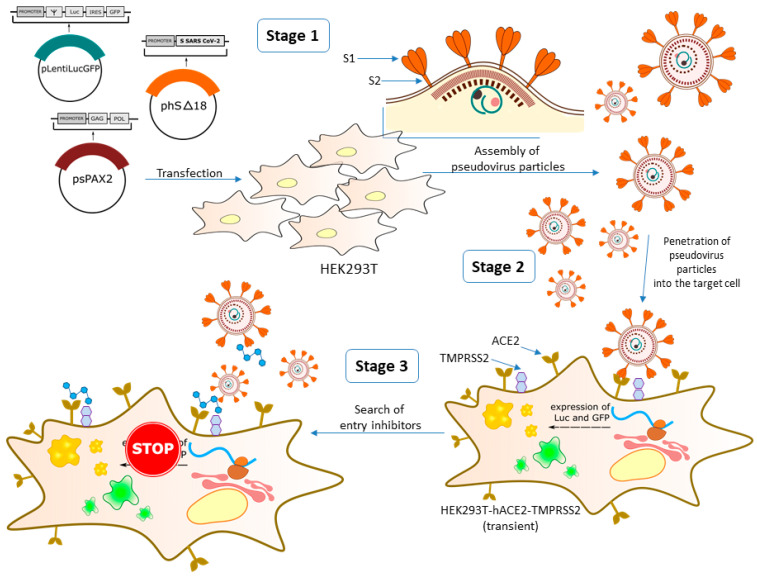
Scheme of work with the pseudovirus system. Experimental work with pseudoviruses includes several stages: (Stage 1) is the assembly of viral particles by transfection of the HEK293 cell line using three plasmids—core, reporter and envelope; (Stage 2) is the determination of the ability of pseudoviral particles to infect target cells; and (Stage 3) is the direct neutralization assay using immune sera or chemotherapeutic agents to determine their ability to block pseudoviruses from entering target cells.

The analysis of potential viral inhibitors using the infectious SARS-CoV-2 virus is associated with the need to comply with a set of BSL-3 biosafety level requirements. The limited number of such laboratories, low research speed, and high cost of work require the search for alternative ways of screening studies. An effective alternative tool to search for penetration inhibitors is the technology of pseudotyped viruses displaying SARS-CoV-2 protein on their surface (Figure 1). Pseudotyped viruses can accurately reproduce the mechanism of SARS-CoV-2 cell penetration and are widely used to study the mechanism of virus penetration, cellular tropism, as well as for viral neutralization assays. Pseudotyped viruses have two distinct advantages over live SARS-CoV-2 virus. First, the pseudotyped virus allows analysis in a conventional BSL-2 laboratory. Secondly, pseudotyped viruses carry marker genes (e.g., luciferase or GFP), allowing for easier and more accurate quantification than if the assay were performed with live SARS-CoV-2 virus. Several viral platforms such as mouse leukemia virus, vesicular stomatitis virus and human immunodeficiency virus are used to obtain pseudotyped SARS-CoV-2 viruses. At the same time, many papers note a good correlation between the experimental results obtained between live SARS-CoV-2 virus and pseudotyped viruses [20].

The aim of the presented work was to find new inhibitors of SARS-CoV-2 virus entry using the pseudoviral systems we developed that have on their surface the S glycoproteins of Wuhan and Delta virus strains. We tested the original libraries of monoterpenoid derivatives, which have previously been shown to have antiviral properties against a wide range of viruses, as the objects of study. In order to search for a possible binding site of the ligands to the surface protein, we tested them on the RBD-ACE2 system. Substances that showed activity against the pseudoviral system were further tested by us using three strains of the infectious virus SARS-CoV-2. As a result of the study, we found that the agents, borneol derivatives, are active against a wide range of SARS-CoV-2 viruses. Molecular modeling of the leader compounds of the putative glycoprotein binding site was performed.

## 2. Materials and Methods

### 2.1. Biological Experiments

#### 2.1.1. Cell Cultures

The HEK293T cell line was provided by the Department “Collections of Microorganisms” of the Rospotrebnadzor State Research Center Vector (Koltsovo, Russia). The HEK293-hACE2 cell line was provided by the Institute of Molecular and Cellular Biology SB RAS (Novosibirsk, Russia). The HEK293T-hACE2-TMPRSS2 (transient) was obtained by transfecting 293 cells with the pDUO-hACE2-TMPRSS2 plasmid. Cells were cultured on Dulbecco’s Modified Eagle Medium (DMEM) (Invitrogen) and Dulbecco’s Modified Eagle Medium/Nutrient Mixture F-12 (DMEM/F12) (SRC Vector, Russia), with the addition of 10% (*v*/*v*) thermally inactivated foetal cow serum (Invitrogen, Carlsbad, CA, USA), and 0.6 mg/mL L-glutamine (Invitrogen, Carlsbad, CA, USA) and 50 µg/mL gentamicin.

Vero E6 cells were used in the experiment (cells of the renal epithelium of an African green monkey) (collection of SRC VB “Vector” Rospotrebnadzor, RF). The cells were cultured in a DMEM medium (Gibco) with L-glutamine, with 10% fetal calf serum (Gibco) and antibiotic-antimycotic (Gibco) at 37 °C, 5% CO_2_.

#### 2.1.2. Plasmids

A second-generation lentiviral system was used to generate pseudoviruses. The psPAX2, which provides formation of lentiviral particles (Addgene #12260), was used as a packaging plasmid. The Ph-SΔ18 encoding the SARS-CoV-2 protein was used as the envelope plasmid and was obtained by inserting the nucleotide sequence encoding the S protein SARS-CoV-2 (GenBank:MN908947) into the phMGFP vector. The last 18 amino acids of the S protein sequence were deleted, and then the codon composition was optimised using the GeneOptimizer tool (https://www.thermofisher.com/ru/en/home/life-science/cloning/gene-synthesis/geneart-gene-synthesis/geneoptimizer.html (accessed on 20 February 2020)). The final nucleotide sequence was synthesized by DNA-Synthesis LLC. The insertion was performed at the NheI and AsiGI sites. In addition, a D614G mutation was introduced into the amino acid sequence of the S protein. The reporter plasmid pLenti-Luc-GFP was obtained from the lentiviral vector pCDH-EF1a-GaussiaSP-MCS-IRES-copGFP (kindly provided by T.N. Belovezhets (ICBFM SB RAS)) by replacing the Gaussia luciferase sequence with that of firefly luciferase. For this purpose, PCR amplification of the firefly luciferase nucleotide sequence was performed using the primers Lenti-Luc-F 5′-aaaaaatctagctagccaccatggaagatgcca-3′ and Lenti-Luc-R 5′-aaaaaaggatccttacacggcgatcttgccg-3′. Plasmid pCAG-luciferase (Addgene #55764) was used as a matrix. Next, the PCR product was inserted into the pCDH-EF1a-GaussiaSP-MCS-IRES-copGFP plasmid at the XbaI and BamHI restriction sites. The pDUO-hACE2-TMPRSS2 plasmid was purchased from the commercial firm Invivogen (San Diego, CA, USA).

#### 2.1.3. Preparation of SARS-CoV-2 Pseudotyped Lentiviral Particles

To obtain SARS-CoV-2 pseudotyped lentiviral particles, we co-transfected HEK293T cells in T75 matrices with three psPAX2 (10 µg), ph-SΔ18 (Wuhan lineages B) or ph-SDΔ18 (Delta lineage B.1.617.2) (10 µg), and pLeni-Luc-GFP (10 µg) plasmids in a 1:1:1 ratio. Lipofectamine 3000 (2 μL per μg of plasmid) (Invitrogen, Carlsbad, CA, USA) was used as a transfectant. The transfected HEK293T cells were incubated at 37 °C in an atmosphere of 5% CO_2_ for two days, after which the supernatant containing lentivirus particles coated with SARS-CoV-2 protein were collected and filtered through a 0.45 μm filter (Millipore, Burlington, MA, USA) and then concentrated on the sucrose pillow. After concentration, 500 µL aliquots were made and stored at −80 °C.

#### 2.1.4. Pseudovirus Entry Assay

Fifty μL of trypsinised suspension of HEK293T cells, HEK293T-hACE2 cells and HEK293T-hACE2-TMPRSS2 (transient) at a concentration of 1 × 106 cells/mL medium was added to a 96-well plate. Then, 10 μL of pseudovirus-containing supernatant was added to the cells in four replicates. After 48 h, the luminescence level was determined using the Luciferase Assay System (Promega, Madison, WI, USA). After the growth medium was removed from the pseudovirus-infected cells, they were lysed using 1 × cell culture lysis buffer (50 μL/well) (Promega). Next, 35 μL of lysate was transferred into black optical plates and the luminescence level was measured using a Varioskan LUX instrument (Thermo Scientific, Waltham, MA, USA) with the automatic addition of luciferase substrate (35 μL/well).

#### 2.1.5. Determination of Cytotoxicity of Compounds on HEK293T Cells

To determine the cytotoxic concentration of compounds, the day before compounds were added to 96-well culture plates, HEK293T cells were seeded in an amount of 100 μL cell suspension per well (104 cells per well) and placed in a CO_2_ incubator. The next day, after 24 h of incubation, different concentrations of test compounds were added to the cell culture by sprouting (initial concentration of 1 mg/mL). Each concentration was tested in three replicates. DMSO at a concentration of no more than 1% was added to the control wells. The final volume of medium in the well was 200 µL. The plate with added compounds was incubated in a CO_2_ incubator for 72 h at 37 °C and 5% CO_2_. After 72 h of incubation of the cell line with the tested compounds, 20 µL of MTT working solution (5 mg/mL) was added to each well and incubated for another 2 h under CO_2_ incubator conditions. After 2 h, plates were removed from the CO_2_ incubator, and the medium in each well was replaced with DMSO solution (50 µL/well). The plates were gently shaken to dissolve the formazan crystals. The optical density of each well at 570 nm was determined using a plate reader. The survival of HEK293T cells in the presence of the test substance was calculated using the formula: (OD of experimental wells − OD of medium)/(OD of control wells − OD of medium) × 100%, where OD is the optical density. The concentration causing 50% cell death (CC_50_) was determined from dose-dependent curves using GraphPad Prism 6 software (Ver. 6.04). For each compound tested for antiviral activity, a range of non-toxic concentrations was selected. A range of nontoxic concentrations, where antiviral activity was investigated, was chosen for each compound.

#### 2.1.6. Determination of Semi-Inhibitory Concentrations of Compounds against Lenti-S SARS-CoV-2 Pseudoviruses and Calculation of Selectivity Index (SI) Values

To determine the inhibitory capacity of the compounds tested, a neutralization assay was performed using HEK293T-hACE2-TMPRSS2 cells (transient) and lentiviral particles exhibiting S protein of SARS-CoV-2. Briefly, serial dilutions of the compounds in DMEM culture medium (without serum or antibiotic) were prepared in 96-well plates. The suspension of pseudoviruses (50 μL/well) was then added to the diluted compounds, and the mixture of compounds with pseudoviruses was incubated in a CO_2_ incubator for 1 h at 37 °C and 5% CO_2_. After 1 h, HEK293T-hACE2-TMPRSS2 cells (transient) (1.5 × 10^4^ cells/well) were added and incubated at 37 °C for 48 h. The assay was performed in three replicates. The infectivity of pseudoviruses in the presence of inhibitors and in control (uninhibited) samples was determined by the luminescence index 48 h after infection. The percentage of neutralization of each sample was calculated as the ratio between the RLU values of the test wells (test sample + pseudovirus + cells) and the virus control (pseudovirus + cells). Statistical data processing and IC_50_ calculations were performed in GraphPad Prism 6 software (Ver. 6.04) using the nonlinear regression method. After IC_50_ was determined, the selectivity index (SI), the ratio of compound toxicity and inhibitory activity against the virus (CC_50_/IC_50_), was calculated (Table 1).

#### 2.1.7. ELISA-Based Competitive Inhibition of the RBD/ACE2 Interaction

In the wells of 96-well plates, RBD protein was adsorbed at a concentration of 400 ng/well in 0.01 M phosphate-buffered saline (PBS, pH 7.2), incubated for 18 h at +4 °C. The plates were then washed with a PBST buffer (0.1% Tween-20 in PBS) and blocked with 1% casein in PBST for 1 h at 37 °C. Solutions of the compounds in DMSO (at a concentration of 10 mg/mL) diluted with phosphate-buffered saline to a concentration of 500 μg/mL were added to the wells and incubated for 1 h at 37 °C. After washing three times with PBST, recombinant ACE2 labeled with horseradish peroxidase was added at a dilution of 1/500 and incubated for 1 h at 37 °C. The wells were washed again and the TMB substrate solution was added. After 15 min, the reaction was stopped with 50 μL of 1 M HCl and absorbance was measured at 450 nm using a Varioskan Lux multi-mode microplate reader (Thermo Fisher Scientific Inc.).

#### 2.1.8. Evaluation of the Antiviral Activities againts SARS-CoV-2 Viruses

Studies using the SARS-CoV-2 virus have been performed in laboratories with BSL-3 containment. The study was carried out using following three coronavirus strains: SARS-CoV-2 nCoV/Victoria/1/2020 (GISAID ID: EPI_ISL_406844, lineages B), hCoV-19/Russia/PSK-2804/2021 (GISAID ID: EPI_ISL_7338814, lineages B1.617.2), and hCoV-19/Russia/Moscow171619-031221/2021 (EPI_ISL_8920444, lineages B.1.1.529) (State collection of pathogens of viral infections and rickettsioses of SRC VB “Vector” Rospotrebnadzor, RF).

The viruses were grown in a Vero E6 cell culture. The infectivity titer of the virus stock was measured at 7.0, 6.5 at 5.5 lgTCD_50_/mL for strains nCoV/Victoria/1/2020, hCoV-19/Russia/PSK-2804/2021 at hCoV-19/Russia/Moscow171619-031221/2021, respectively. Vero E6 cells were grown in 96-well culture plates to a confluence of at least 95%. Samples were dissolved in dimethyl sulfoxide (DMSO) to a concentration of 10 mg/mL. Remdesevir was used as a control drug. Evaluation of the effective (IC_50_) concentrations of compounds was carried out in the test to reduce the cytopathic effect on cells. Serial three-fold dilutions of the compounds were prepared, starting at a concentration of 600 μg/mL. To test each compound, the virus doses of 100 and 10 TCD_50_ per well were used.

Inhibitory activities and toxicities of the tested compounds were assessed simultaneously. Specifically, dilutions of the compounds were added to the wells in the culture plates containing a monolayer of cells. Plain medium (to determine the toxic concentration of the tested compounds) or medium containing a virus (to determine inhibitory activities) were then added. The culture plates were incubated at 37 °C for 4 days; after which they were stained using the MTT assay protocol. The results were recorded with ThermoScientificMultiskanFC; data processing was carried out in the SOFTmax PRO 4.0 program using a 4-parameter analysis method. The 50% toxic concentration (CD_50_) and the 50% inhibition (IC_50_) concentrations were both determined. The selectivity index (SI) as the ratio of the compound toxicity and inhibitory activity was also calculated (Table 2).

#### 2.1.9. Statistical Analysis

All statistical analyses were performed using GraphPad Prism software, San Diego, CA, USA.

### 2.2. Synthesis of Compounds

Details of synthesis of compounds 1, 2 and 4, 5 are present in the work [21]. Synthesis of compound **3** is described in [22]. Syntheses and structural characterization of the (-)-borneol esters **6**, **7**, **11**–**14**, **16**, **17**, **21** [23] and esters **8**–**10**, **15**, **18**–**20**, **22**–**29** as well as amides **30**–**35** that were described in our previous publications [24,25]. Details of compound synthesis are found in the Supplementary Materials document.

### 2.3. Molecular Dynamics and Docking

#### 2.3.1. Ligand and Protein Preparation

The SARS-CoV-2 S-protein structure model was taken from the RCSB Protein Data Bank [26]. We used three variations of the S-protein structure for calculations: 7BNM [27] original Wuhan virus strain, 7W94 [28]—Delta B.1.617.2, 7QO7—Omicron B.1.1.529 [29]. All SARS-CoV-2 S-protein structure models were preprocessed with Schrodinger Protein prepwizard tools: added and refined hydrogen atoms (and hydrogen bonds network), added missing amino acid sidechains, verified bond orders, solvent elimination, and restrained minimization of protein structure in OPLS4 forcefield [30]. 

Potential ligands geometry was optimized taking into account all admissible conformations. Protonation of the nitrogen atom, based on pKa values of compounds **11** and **21** was also considered. DFT (level of theory B3LYP [31], with basic set cc-pVTZ(+), [32]) calculations was processed in Jaguar program [33], with the use of an early presented protocol, shown by the authors in previous papers [34].

#### 2.3.2. Possible Ligand Binding Site Search

A possible binding site was selected in accordance with early published modelling results shown in [35,36]. Compound **21** can be located in the hydrophobic cleft, located between the α-helices of protomers. By analogy with Arbidol binding with the influenza virus hemagglutinin and SARS-CoV-2 S-protein, we assumed that three molecules of potential inhibitors could interact there per one trimeric SARS-CoV-2 virus glycoprotein. The following functional amino acids were considered as potential ligand-binding interactants due to the positioning near heptad repeat. They are the residues K776, R1019, N1023, L1024, and T1027. After relaxation of the S-protein trimeric complex, the geometrical parameters of the system were used for the molecular docking procedure in order to estimate the binding energy of compounds **11** and **21** at the putative binding site.

#### 2.3.3. Molecular Docking and Dynamics Simulations

Molecular systems used for MD simulations are based on the S-protein structure in trimeric form, complexed with three ligands respectively, placed in cavities located in the region of heptad repeats. Protein-ligand complexes were placed into orthorhombic box with a buffer zone of 26 Å from the protein surface. The molecular system also includes 0.15 M NaCl aq. solution (saline). The solvent model is TIP3P. Na and Cl ions are used as a counterions neutralizing system. The ensemble NPT (isothermal–isobaric ensemble) was used. Simulation time is 100 ns at T = 310 K (37 °C). The system preparation protocol for simulation includes pre-minimization and equilibration stages: simulation in the NVT ensemble with Brownian dynamics at 10 K with small time steps and solute non-hydrogen atoms restrained; simulation in the NVT ensemble using a Langevin thermostat with simulation time of 12 ps, temperature of 10 K, resampling of fast temperature relaxation constant velocity every 1 ps, non-hydrogen solute atoms restrained; simulation in the NPT ensemble using a Langevin thermostat and a Langevin barostat with simulation time of 12 ps, temperature of 10K and a pressure of 1 atm, fast temperature relaxation constant, slow pressure relaxation constant velocity resampling every 1 ps, non-hydrogen solute atoms restrained; simulation in the NPT ensemble using a Langevin thermostat and a Langevin barostat with simulation time of 12 ps, temperature of 300 K and a pressure of 1 atm, fast temperature relaxation constant, slow pressure relaxation constant, velocity resampling every 1 ps, non-hydrogen solute atoms restrained; simulation in the NPT ensemble using a Langevin thermostat and a Langevin barostat with simulation time of 24 ps, temperature of 300 K and a pressure of 1 atm, fast temperature relaxation constant, and normal pressure relaxation constant. All calculations were performed using Desmond software [37] that is included in Schrodinger Suite 2021-4.

Molecular docking of compounds **11** and **21** was done using the induced-fit docking method with the following conditions at the input: grid size of 15 Å, protein residues in 5 Å-radius around ligand, and restrained optimization in accordance with ligand influence. Docking solution ranking was based on docking score (GlideScore including state penalties), ligand efficiency, Emodel value (includes GlideScore and (for flexible docking) the internal strain energy for the model potential used to direct the conformational-search algorithm) and binding poses clustering. Binding free energy (ΔG, kcal/mol) of compounds **11** and **21** in possible binding site was evaluated using molecular mechanics with the generalized Born and surface area (MM-GBSA) methods [38]. Solvent (water) was included as an implicit model. The Glide IFD [39] and Prime [40] programs from Schrodinger Suite 2021-4 were used for calculations.

For the resulting trajectories, a population analysis was carried out using the VolMap plugin implemented in the VMD program [41]. Two types of maps based on weighted atomic density and weighted atomic population were considered to determine the statistical binding site of ligands **11** and **12** to the protein. The smaller the cloud size, the lower the probability of finding the molecule in that region of space. To quantify protein structure changes before and after simulation, the Simulations interactions diagram tool from Schrodinger Suite 2021-4 was used. The RMSD parameter was chosen as the change descriptor. The diagrams of RMSD and protein-ligand interactions were calculated for each ligand (three ligands per system).

## 3. Results and Discussion

### 3.1. Development of a Pseudovirus System

**Figure 2:**
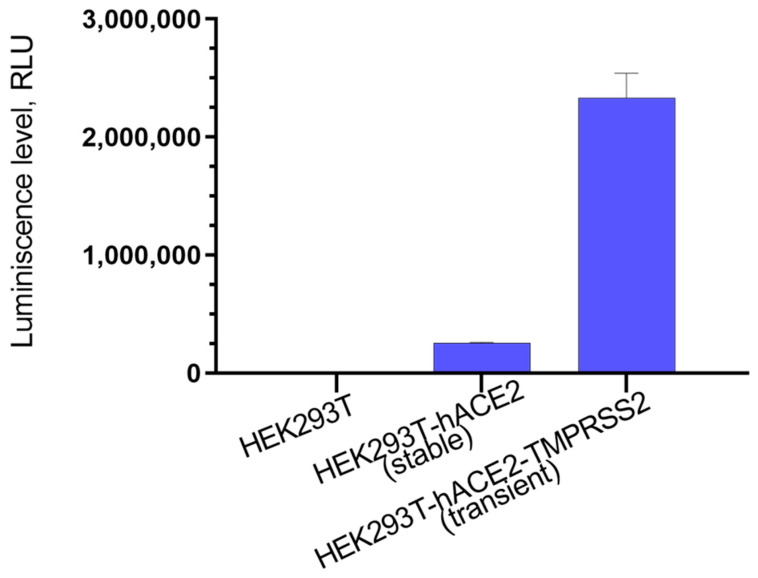
Infectivity of pseudoviral particles containing the SARS-CoV-2 protein on the S surface in HEK293T, HEK293T-hACE2 (stable) and HEK293T-hACE2-TMPRSS2 (transient). At 48 h post-infection, pseudovirus entry was analyzed by determining luciferase activity in cell lysates.

To assess the inhibitory activity of synthesized compounds, we used lentiviral particles carrying SARS-CoV-2 protein S on their surface. To obtain lentiviral particles, psPAX2 was used as a packaging plasmid, which ensures the formation of lentiviral particles; ph-SΔ18 plasmid was used as the plasmid containing the surface protein gene. Two versions of the plasmid were used: the first one contained the Wuhan strain S protein gene, and the second one contained Delta B.1.617.2; in both cases a C-terminus region of 18 bp was deleted. The plasmid pLenti-Luc-GFP was used as a reporter plasmid. After assembly and concentration of pseudoviral particles, their functional activity, that is, the ability to infect target cells, was evaluated. Since it is known that pseudoviruses containing the SARS-CoV-2 S protein on their surface either do not infect the original HEK293T cells at all, or infect them with low efficiency [34], we used HEK293T-ACE2 (s) stably exhibiting angiotensin-converting enzyme 2 (ACE2) on its surface, since the SARS-CoV-2 S protein is known to interact with ACE2 as its target [35]. Additionally, HEK293T-hACE2-TMPRSS2 (t) cells obtained transiently and containing both hACE2 and TMPRSS2 on their surface were used, since it was shown that the viral entry requires priming of the S-protein by cellular proteases, such as the TMPRSS2 serine protease, which ensures fusion of viral and cell membranes [36]. According to the results obtained, the best infectivity of pseudoviral particles is observed for HEK293T-hACE2-TMPRSS2 (t) cells, while on HEK293T-ACE2 (s) cells, our SARS-CoV-2 pseudoviruses had a ten-fold lower infectivity and were almost non-contagious in HEK293T cells (Figure 2).

### 3.2. Choice of Research Objects

**Figure 3:**
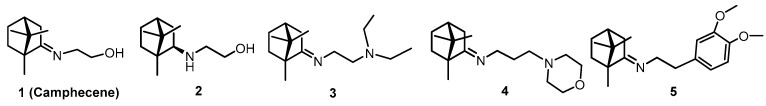
First class compounds—camphor derivatives.

In this work, we tested three classes of compounds using the pseudovirus system that we have developed. All of the substances under study, as we showed earlier, are active against various viruses. Thus, camphor derivatives **1**–**5** are effective inhibitors of influenza virus (Figure 3) [21,22]. The product of the interaction of camphor and aminoethanol, camphecene **1**, has a broad spectrum of antiviral activity against strains of influenza virus of type A H1N1 (swine), H3N2 (Hong Kong), H5N2 (avian) and type B virus [42]. Studies on the effect of camphecene and its analogues on inhibition of influenza virus depending on the time of addition show that the compounds of this series demonstrate the activity at the early stages of viral replication. The camphecene-resistant strains of influenza virus were selected and a mutation in the structure of hemagglutinin that provides the resistance phenotype to the virus was localized [43]. Mutations in the surface hemagglutinin protein in the camphecene-resistant strain are located near the HA2 subunit fusion peptide. We studied the mechanism of this agent action [44] and demonstrated its high efficacy in animal models [45,46].

**Figure 4:**
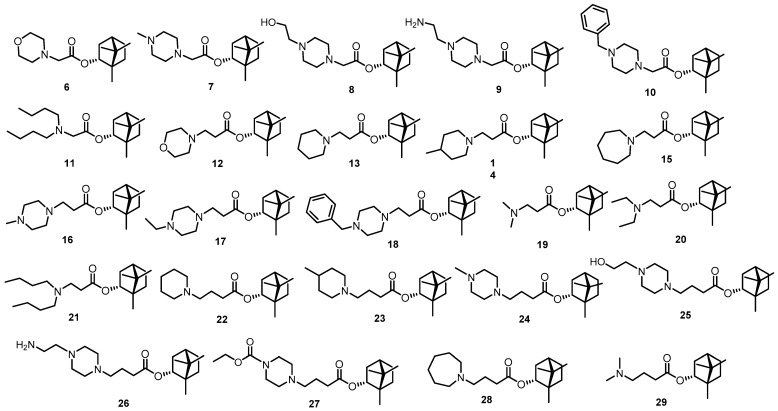
Compounds of the second class—ester derivatives based on (-)-borneol.

Compounds of the second class—ester derivatives based on (-)-borneol **6**–**29**—show their activity against a wide range of envelope viruses (Figure 4). Thus, activity against H1N1 influenza viruses was shown for compounds of this class [23], activity against orthopoxviruses was detected [25,47]; these substances showed particularly high activity against filoviruses Marburg and Ebola [24,48]. We studied the action mechanism of these agents against filoviruses using the method of site-directed mutagenesis and showed that the substances exhibit their activity on the surface protein of the Ebola virus [24]. Compounds **6**–**11** have one CH_2_ group between the terpene fragment and the tertiary nitrogen atom, substances **12**–**21** have a C_2_H_4_ linker and agents **22**–**29** have a C_3_H_6_ linker. All agents contain a tertiary nitrogen atom either in a saturated heterocyclic fragment or with aliphatic substituents.

**Figure 5:**
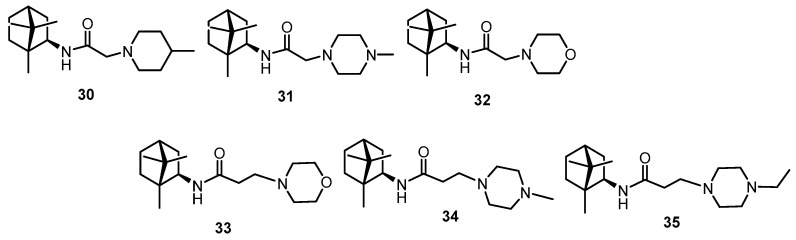
Compounds of the third class—isobornylamine-based amides.

The third class of studied substances was presented by isobornilamine-based amides (Figure 5). The method for isobornylamine synthesis was described earlier [25]. These compounds showed activity against the smallpox virus [25] and against filoviruses [24]. Similarly to the ester derivatives of the second group, substances **30**–**32** have a linker of one CH_2_ group and compounds **33**–**35** of two CH_2_.

All substances were synthesized according to the published methods and had a purity of at least 98%.

### 3.3. Testing with the Use of Pseudo-Viral Systems

We analysed the activity of compounds from three groups using the obtained lentiviral particles carrying the SARS-CoV-2 virus glycoprotein on their surface. The test results are presented in Table 1. We used Arbidol as a reference drug.

The results of our study show that the compounds of the first group—camphor imin derivatives **1**, **3**–**5** and amine **2**—showed no activity at all on the pseudoviral system with SARS-CoV-2 virus glycoprotein on its surface (Wuhan lineages B strain). Among borneol-based ester derivatives, agents **10**, **11**, **12**, **18** and **21** showed activity. Among the compounds of the third class, only substance **30** showed moderate activity.

We then tested all active agents using a pseudovirus system with protein S from the Delta strain (Delta lineage B.1.617.2) on its surface. Only agents **11** and **21** were active, and their IC_50_s were 16.0 and 14.2 μM, respectively. Further efforts were aimed at determining the activity against the infectious virus SARS-CoV-2 and clarifying the mechanism of action.

### 3.4. Antiviral Activity against SARS-CoV-2 Viruses

We studied all substances that showed activity on the pseudovirus system using an infectious virus. Vero E-6 cells were used, and toxicity and inhibitory activity were studied simultaneously. The results are summarized in Table 2. The studies showed that agents **10** and **18** containing the 4-benzyl-piperazine fragment were more toxic on the cell line studied than agents **11** and **21**. Agents **11** and **21** showed the lowest toxicity, and their CC_50_ was over 300 µM.

Two doses of virus 100 TCD_50_ and 10 TCD_50_ were used.

Studies were performed on three strains of the virus: Wuhan, Delta and Omicron. The first SARS-CoV-2 strain, used as a prototype in our experiments, is hCoV-19/Australia/VIC01/2020 lineages B (Wuhan lineages B). Viruses of this genetic lineage circulated during the first wave of the pandemic [49]. Following its spread to the rest of the world, many unique mutations in the virus genome have been reported. The biological advantages conferred by some of these mutations have resulted in the creation of specific genetic lineages that have largely outcompeted the original strain. Lineages that are characterized by increased transmissibility or virulence or that decrease the effectiveness of available countermeasures are classified by the WHO as variants of concern (VOCs). The second SARS-CoV-2 strain is hCoV-19/Russia/PSK-2804/2021, SARS-CoV-2 lineage B.1.617.2 (Delta). The SARS-CoV-2 delta (B.1.617.2) variant was first detected in India in October 2020. It has since rapidly become the predominant lineage, owing to much higher transmissibility. The Delta variant has been associated with an increase in the severity of illness, as illustrated by a higher rate of hospitalization among patients with the Delta COVID-19 variant when compared to the Alpha variant, especially among the unvaccinated ones [50]. The third strain is the Omicron virus hCoV-19/Russia/Moscow171619-031221/2021 lineage B.1.1.529. The B.1.1.529 variant was first reported to the WHO from South Africa on 24 November 2021 [51]. The Omicron virus strains are responsible for a significant increase in incidence in late 2021 and January-February 2022 [52].

The studies presented showed that only agents **11** and **21** showed activity against infectious viruses. Thus, against the Wuhan strain, compound **11** showed higher activity than its analog with a longer linker, agent **21**. Using a virus dose of 100 TCD_50_, the semi-inhibitory concentration for these agents is 13.0 and 51.1 µM, respectively. When the dose of the virus is lowered, the amount of substance decreases significantly, and IC_50_ for these agents is 9.6 and 4.7, respectively. Studies on the Delta strain showed that agent **21** exhibited high activity comparable to the reference drug, Remdesivir. The two studied agents also showed high activity on the Omicron strain. In all cases we see a dose-dependent effect. In general, we can conclude that agent **21** is more promising for further studies of the mechanism of action and for studies using an animal model

### 3.5. ELISA-Based Competitive Inhibition of the RBD/ACE2 Interaction

Compounds capable of inhibiting the entry of pseudoviruses into target cells can act through different mechanisms. This may be a direct blocking by substances of binding of the virus surface proteins to their target receptors. Indirect inhibition is also possible, when substances block conformational rearrangements or other important steps in the work of viral fusion proteins. Implementation of the first blocking mechanism is quite easy to detect if the virus has one target for interaction. For SARS-CoV-2, this is possible because the target of this virus is the cellular receptor ACE2. For compounds that had an inhibitory effect on pseudoviruses, we analyzed their ability to inhibit interaction of recombinant ACE2 with RBD. It was shown that none of the substances used in the work was able to inhibit this interaction, so we assume that the studied compounds have an inhibitory effect on the second pathway.

### 3.6. Molecular Modeling Study

#### 3.6.1. Finding and Reasoning about the Binding Site of Potential Entry Inhibitors

**Figure 6:**
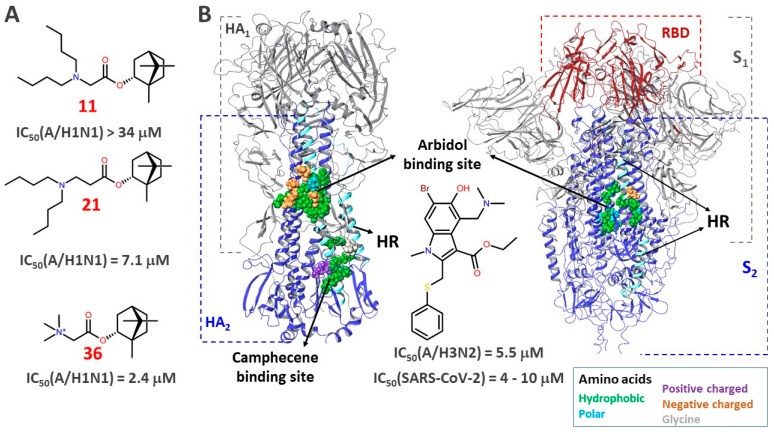
(**A**)—Structures of compounds **11**, **21**, and **36** containing a similar structural fragment. (**B**)—Surface viral proteins hemagglutinin (HA) of influenza virus and glycoprotein (S-protein) SARS-CoV-2. The secondary structures of proteins that make up the first HA1 and S1 subunits are shown in gray. The receptor binding domain (RBD) of the S-protein is shown in red. α-Heptad repeats (HR), make up the second HA2 and S2 subunits. The Arbidol binding sites in both surface proteins are located between the α-helix of the two protomers. The amino acids included in the binding site are colored according to their pharmacophoric signatures. The Camphecene binding site is located at the HA proteolysis site. Surface proteins were visualized based on the PDB [26] codes of HAs of the A/H1N1 4LXV strain [61] and the SARS-CoV-2 6VXX S-protein [62].

Biological experiments allowed us to identify two leader compounds, **11** and **21**, among a number of borneol derivatives. The mechanism of their antiviral activity is probably related to the inhibitory effect on the SARS-CoV-2 surface protein. This is evidenced by the results of the evaluation of the activity of these compounds in pseudovirus experiments. Molecular modeling methods are designed to determine the potential binding site of leader compounds on the surface protein of the coronavirus. Most researchers using the theoretical approach consider its receptor-binding domain (RBD), in particular the RBD binding interface with ACE2, as a likely binding site for low molecular weight S-protein inhibitors. The assumption that binding of a small molecule to the RBD can attenuate to a large extent the interaction of the protein domain with the cellular enzyme seems to be a very sound strategy in the search for drugs. However, in our case, the results of the RBD-ACE2 competitive inhibition assay experiment demonstrate that the agents under investigation have no effect on RBD binding to ACE2. The obvious solution is to search for the binding site of potential entry inhibitors specifically in the stem portion of the S2 subunit of the SARS-CoV-2 glycoprotein (Figure 6).

We have previously shown that agents **11** and **21** (Figure 6A) exhibit anti-viral activity against influenza virus of strain A/H1N1 [23]. It was further shown in [53] that a number of borneol derivatives, among which compound **36**, having a common structural fragment (bicyclo[2.2.1]norbornane) with compounds **11** and **21**, inhibit the fusogenic activity of hemagglutinin (HA). It has been suggested that the compounds can bind at the stem portion of HA: at the fusion peptide site, the so-called TBQH or Arbidol binding site [54] and/or at the proteolysis site, the Camphecene binding site [43,44]. Both proposed HA inhibitor binding sites are located in the second subunit in the heptad repeats (HR) region. In other words, compounds **36**, **11**, and **21** can be considered as inhibitors of influenza virus entry.

The HA surface proteins and the SARS-CoV-2 S-protein belong to type I of the viral fusion proteins, with a common mechanism of viral and cell membrane fusion [55]. Both proteins play a key role related to the entry of the virus into the host cell. Like HA, the S-protein consists of two functional domains, S1 and S2 (Figure 6B). The role of S1 is to bind the receptor-binding domain RBD (shown in red in Figure 6B), located in the C-terminal subdomain, to ACE2. Subsequent cleavage of the glycoprotein by cellular proteases exposes the fusion peptide or S2 domain. The conformational rearrangements in S2 lead to the fusion of the viral and cell membranes, as a result of which the virus delivers its genome into the cell cytoplasm [56]. Heptad repeats (HR), which are in a compressed state until activation (lowering the pH of the medium), take part in the conformational rearrangements. Stabilization of the “compressed” or pre-fusion state by small molecules can prevent conformational transitions and both inhibit viral and cell membrane fusion. Some HA inhibitors work in a similar way [57], including Arbidol [58]. The presence of similar heptad repeats in HAs and S-protein suggests similar hydrophobic cavities (hydrophobic amino acids are shown by green spheres in Figure 6B) in the space between the spirals of the stem part of the protein. It is also assumed that Arbidol, which exhibits moderate antiviral activity against SARS-CoV-2 in in vitro tests [59,60], binds exactly in the stem portion of the S-protein domain [35,36] (Figure 6B).

All of these considerations together with the data from biological experiments allow us to consider the surface SARS-CoV-2 protein as a potential biological target explaining the mechanism of antiviral activity of compounds **11** and **21**.

**Figure 7:**
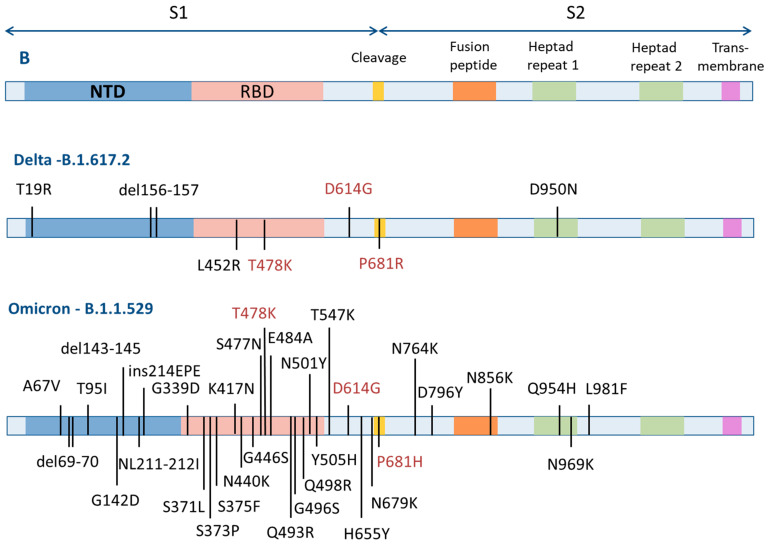
Mutations of amino acid residues in different strains of the SARS-CoV-2 virus considered in this study.

The binding site is most likely located in the second S2 subunit, in the region of the heptad repeats (HR1). This is supported by the following fact: the antiviral activity of **11** and **21** on different strains of the virus is commensurate. At the same time, it is well known that during the ongoing pandemic of coronavirus infection there is adaptation of the virus to the human host [63] as a result of mutations of amino acid sequences specifically in the surface S-protein (Figure 7). Multiple mutations occur in S1 in the NTD and RBD domains, while in the second subunit they are axial in nature.

Thus, the results of biological experiments, the structural features of the studied compounds, and the analysis of the amino acid sequence of the SARS-CoV-2 surface protein allow us to assume that compounds **11** and **21** bind in the stem portion of the S2 domain. The mechanism of antiviral activity of the studied structures probably consists in the suppression of the fusogenic activity of the surface protein.

#### 3.6.2. Protonation of Ligands

**Figure 8:**
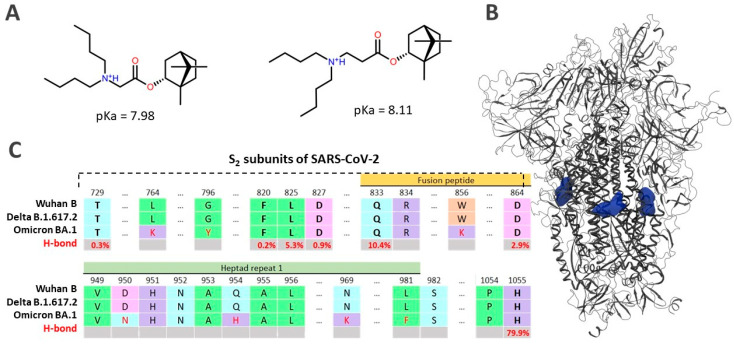
(**A**)—is the result of the estimation of the acidity constant of the leader compounds by quantum chemistry; (**B**)—is the weighted atomic population map of compound **21** based on the results of molecular dynamic simulations; (**C**)—is the comparison of a number of amino acid residues of part S2 of SARS-CoV-2 of various strains, and replacements are shown in red font; H-bond is the frequency of formation (in %) of hydrogen bonds between ligand atoms and amino acids.

In all theoretical calculations of this work, the possibility of protonation of the nitrogen atom in compounds **11** and **21** was taken into account. Protonation of the nitrogen atom creates an additional reaction binding site in the molecule preferable for the formation of hydrogen and/or salt bridges. According to the results of quantum-chemical calculations, the values of acidity constant pKa estimated by the DFT methods for compounds **11** and **21** are 7.98 and 8.11, respectively (Figure 8A). In other words, the compounds are in the protonated state at the tertiary nitrogen atom both at physiological and at lower pH values of the medium.

#### 3.6.3. Molecular Dynamics Simulation

Analysis of the results of molecular dynamics simulations allows us to specify a possible binding site for potential S-protein inhibitors of SARS-CoV-2. The starting location of compound **21** was created based on the results of molecular dynamics of the S-protein system—3 Arbidol molecules located in the region of central and heptad repeats between the two protomers of the protein [35,36]. Just as in the case of Arbidol, there are three potential inhibitor molecules per S-protein of SARS-CoV-2.

Analysis of the RMSD system showed that the ligand-protein complex had already reached equilibrium at 40 ns of simulation (Supplementary Materials, Figure S1). The compounds form a series of stable intermolecular interactions with amino acids (Figure S2): hydrogen bonds, including those mediated by water, and salt bridges. The hydrophobic part of the molecule most often contacts a number of hydrophobic amino acids, such as valine, leucine, alanine, and isoleucine, throughout the simulation time (Figure S3). The most significant among these are the contacts with Asp827/864, Gln833, and His1055. Contacts with the lipophilic amino acid residues Ala1053/826, Val949/726, Phe830/820 and Leu825 in turn become more intensive with simulation time. This fact suggests a specific retention of the ligand in the active site. Analysis of the weighted atomic occupancy map allows for the determination of the probable position of ligand molecules throughout the simulation time and frequency of formation of hydrogen bridges with certain amino acids. Then, according to the map (Figure 8B), each molecule is located in a separate protomer, between the α-helix of the central part of S2.

Figure 8C shows the alignment of the amino acid residues included in the probable binding site of different SARS-CoV-2 strains. The evolution of the virus here is represented as point mutations. A molecular dynamics simulation analysis shows that hydrogen bonds are formed between compound **21** atoms and conserved amino acids such as Thr729, Phe820, Leu825, Asp827/864, Gln833, and His1055. The frequency of hydrogen bonds with these amino acids formed throughout the simulation time is shown in red in Figure 8C. The fact that the antiviral activity of compounds **11** and **21** on the strains studied is approximately comparable can be considered as additional evidence of the binding site.

#### 3.6.4. Molecular Docking

**Figure 9:**
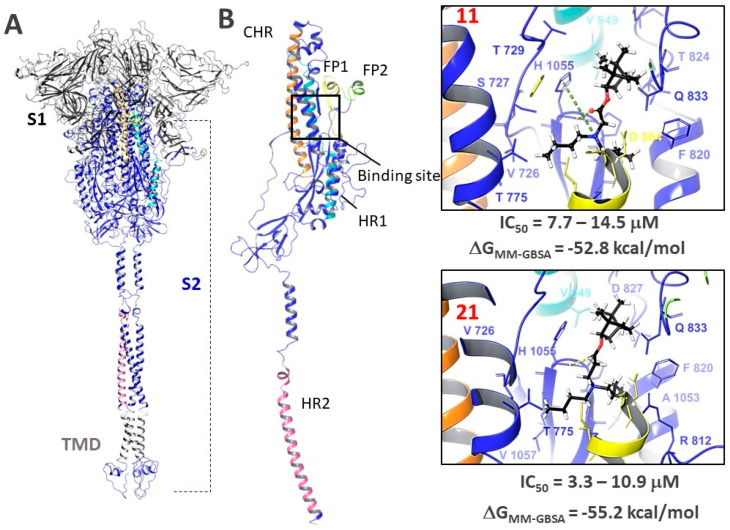
(**A**)—full-length visualization of the surface protein: S1 and S2 surface protein subunits, TMD—transmembrane domain, CHR, HR1, HR2—central and heptad repeats 1 and 2, respectively, FP1, FP2—fusion peptides; (**B**)—location of agents **11** and **21** in the supposed binding site: hydrogen bonds are shown with a yellow dashed line, π-cation interaction—with a green dashed line. The ranked IC_50_ values illustrate the antiviral activity against different SARS-CoV-2 virus strains.

Based on the results of molecular dynamic simulations, we hypothesize that the fusion inhibitor binding site is probably located in the central repeat (CHR) and heptad repeats (HR1) region. This region is located in the second subunit of the S-protein, the most conserved part of the protein (Figure 9A), as compared to S1. The binding site is saturated with hydrophobic amino acids such as alanine, leucine, valine, and proline. Molecular IFD docking allowed us to choose the most favorable positions in terms of ligand-protein interaction of energy parameters. Compounds **11** and **21** are located in the binding site with the formation of a hydrogen bond with His1055. Additionally, **11** forms a cation-π stacking interaction between the protonated nitrogen atom and the aromatic ring of His1055. In all cases, hydrophobic interactions are observed between the lipophilic parts of the inhibitors (the bicyclo 2.2.1 framework fragment and butyl substituents) and the hydrophobic amino acids of the binding site. The binding energies (ΔG_MM-GBSA_) of both ligands in the binding sites are comparable, which correlates with the results of biological experiments.

Thus, based on the results of large-scale molecular modeling combined with the results of biological experiments, we suggest that compounds **11** and **21** can bind in the hydrophobic spaces between the α-helix of the S2 subunit of the glycoprotein, thereby suppressing the fusogenic activity of the protein.

Summarizing the presented study, we should pay attention to the following perspective. Thus, various physicochemical mechanisms play an important role in the processes of cell infection by enveloped viruses. For example, the key stage of SARS-CoV-2 penetration into the cell is the fusion of its envelope with the cell plasma membrane. An important role in this process is played by the fusion peptides of its surface S proteins, which penetrate into the lipid matrix of the cell membrane and bring it into contact with the viral envelope to realize fusion. Much attention is currently being paid to studying the structure of the receptor-binding domain of protein S. However, the emergence of new strains of SARS-CoV-2 coronavirus suggests the high variability of this site, which reduces the effectiveness of vaccines and antiviral drug candidates targeting this element of the S protein. At the same time, studies have shown that the SARS-CoV-2 fusion peptide contains fairly conserved sites that are characteristic not only of this virus, but also of the entire family of beta-coronaviruses. Targeting the action of low molecular weight substances on the conserved fragments of surface glycoproteins may be a more promising strategy to search for effective anticoronoviral agents with a wide spectrum of action.

## 4. Conclusions

In this work, we constructed a pseudoviral system with the surface glycoproteins of SARS-CoV-2 viruses of two lines: Wuhan and Delta. In order to search for entry inhibitors, we chose three classes of monoterpenoid derivatives that had previously shown antiviral activity against various viruses as objects of study. The results showed that among the ester compounds—bicyclic alcohol (-)-borneol derivatives—we found the compounds that showed a pronounced activity against the pseudovirus system. For the agents selected in the first stage, testing was performed using three infectious strains of the SARS-CoV-2 virus: Wuhan, Delta, and Omicron. Independent experiments showed that agents **11** and **21** with in a bornane backbone and dibutyl-substituted tertiary nitrogen atom in their structure show high activity against all three virus strains; this activity is comparable with that of the comparison drug, Remdeservir. In order to identify the site of ligand binding to the surface protein, we additionally analyzed competitive inhibition of RBD-ACE2. It was shown that the studied substances do not affect the binding of RBD-ACE2, which allowed us to limit the search for potential binding sites.

Since compounds **11** and **21** were previously shown to be active against the influenza virus and given that they act at an early stage of viral replication, we compared the influenza and SARS-CoV-2 glycoproteins. The presence of similar heptad repeats in the HA and S-protein suggests similar hydrophobic cavities in the space between the α-helices of the stem part of the protein. The binding site is most likely located in the second subunit of S2, in the region of heptad repeats (HR1). This is supported by the commensurate antiviral activity of agents **11** and **21** on different strains of the virus. In other words, the results of biological experiments, structural features of the studied compounds and analysis of the amino acid sequence of the surface SARS-CoV-2 protein suggest that compounds **11** and **21** bind in the stem portion of the S2 domain. Based on the results of molecular dynamic simulations, we suggest that the fusion inhibitor binding site is probably located in the central repeat (CHR) and heptad repeats (HR1) region. This region is located in the second subunit of the S-protein, the most conserved part of the protein compared to S1. In all cases, hydrophobic interactions are observed between the lipophilic parts of the inhibitors (the bicyclo 2.2.1 framework fragment and butyl substituents) and the hydrophobic amino acids of the binding site.

Based on in vitro results with an infectious virus, in a pseudovirus system and theoretical calculations, we believe that compounds containing a bornane backbone and a tertiary nitrogen atom in their structure can be considered as scaffold structures. Such compounds can be structurally modified to create novel entry inhibitors active against a wide range of SARS-CoV-2 strains. We believe that the strategy of finding low-molecular-weight inhibitors that bind to conserved sites of surface proteins is a prospective direction.

## Figures and Tables

**Table 1:** Inhibitory activity against pseudoviruses pseudotyped with S protein of Wuhan lineages B and Delta lineage B.1.617.2.

Compound	CC_50_ ^a^	Wuhan Lineages B		Delta Lineage B.1.617.2	
	µM	IC_50_ ^b^ µM	SI ^c^	IC_50_ ^b^ µM	SI ^c^
**1**	2950	>500	-	NT ^d^	−
**2**	1260	>500	-	NT	−
**3**	1260	>500	-	NT	−
**4**	1080	>440	-	NT	−
**5**	660	>200	-	NT	−
**6**	982	188.6 ± 21.1	5	NT	−
**7**	1455	>500	-	NT	−
**8**	1045	>500	-	NT	−
**9**	346	>200	-	NT	−
**10**	362	15.6 ± 2.1	23	>100	−
**11**	186	17.7 ± 3.2	10	16.0 ± 4.5	11
**12**	396	21.3 ± 4.3	18	>100	−
**13**	960	>200	-	NT	−
**14**	540	>200	-	NT	−
**15**	538	>200	-	NT	−
**16**	350	>200	-	NT	−
**17**	855	>200	-	NT	−
**18**	292	17.7 ± 3.7	17	>100	−
**19**	1015	>200		NT	−
**20**	935	>200		NT	−
**21**	743	25.8 ± 4.2	29	14.2 ± 2.9	53
**22**	407	>200	-	NT	−
**23**	350	>200	-	NT	−
**24**	348	155	2	NT	−
**25**	438	196	2	NT	−
**26**	179	59.8 ± 7.1	3	NT	−
**27**	166	52.6 ± 8.3	3	NT	−
**28**	1080	190	5	NT	−
**29**	490	>200	-	NT	−
**30**	417	41.2 ± 5.2	10	>100	−
**31**	1525	156	9	NT	−
**32**	820	>200	-	NT	−
**33**	2160	>200	-	NT	−
**34**	1140	>200	-	NT	−
**35**	1260	190	6	NT	−
**Arbidol**	14.8	7.8 ± 3.1	2	NT	−

^a^ CC_50_ is the cytotoxic concentration, the concentration resulting in the death of 50% of cells; ^b^ IC_50_ is the 50% virus-inhibiting concentration, the concentration leading to 50% inhibition of virus replication; ^c^ SI is the selectivity index, the ratio of CC_50_/IC_50_. The data presented are the mean of three independent experiments. The values for CC_50_ and IC_50_ are presented as the mean ± error of the experiment. ^d^ NT: no testing.

**Table 2:** Antiviral activities of derivatives **10**, **11**, **12**, **18** and **21** against SARS-CoV-2 virus strains obtained on Vero E6 cell culture.

Agent		Wuhan Lineages B ^a^		Delta Lineage B.1.617.2 ^b^		Omicron Lineage B.1.1.529 ^c^	
	CC_50_ ^d^_,_ µM	IC_50_ ^e^, µM	SI ^f^	IC_50_, µM	SI	IC_50_, µM	SI
**10**	110.5 ± 11.2	NA	-	NA	-	NA	-
**11**	313.5 ± 14.1	13.0 ± 2.1 *	24 *	28.2 ± 3.1 *	11 *	14.5 ± 2.1 *	22 *
		9.6 ± 1.4 **	32 **	17.6 ± 2.1 **	17 **	7.7 ± 1.8 **	40 **
**12**	261.3 ± 16.3	NA	-	NA	-	160.5 ± 18.2 *	1.6 *
**18**	103.8 ± 13.1	NA	-	NA	-	NT	-
**21**	336.6 ± 16.6	51.1 ± 6.2 *	6 *	10.9 ± 1.8 *	30 *	10.9 ± 1.5 *	30 *
		4.7 ± 6.2 **	71 **	3.5 ± 6.2 **	96 **	3.3 ± 6.2 **	102 **
Remdesivir	710.9 ± 21.2	13.7 ± 2.2 *	51 *	7.3 ± 1.5 *	97 *	6.6 ± 1.1 *	107 *
		3.8 ± 0.42 **	186 **	2.1 ± 0.16 **	338 **	2.0 ± 0.13 **	356 **

^a^ hCoV-19/Australia/VIC01/2020 (EPI_ISL_406844); ^b^ hCoV-19/Russia/PSK-2804/2021 (EPI_ISL_7338814); ^c^ hCoV-19/Russia/Moscow171619-031221/2021 (EPI_ISL_8920444); ^d^ CC_50_ is the cytotoxic concentration, the concentration resulting in the death of 50% of cells; ^e^ IC_50_ is the 50% virus-inhibiting concentration, the concentration leading to 50% inhibition of virus replication; ^f^ SI is the selectivity index, the ratio of CC_50_/IC_50_. The data presented are the mean of three independent experiments. The values for CC_50_ and IC_50_ are presented as the mean ± error of the experiment.; * 100 TCD_50_; ** 10 TCD_50_.

## Data Availability

Not applicable.

## Supplementary Materials

The following supporting information can be downloaded at: https://www.mdpi.com/article/10.3390/v14061295/s1, Figure S1: System RMSD during simulation; Figure S2: Ligand-protein interactions percentage and type; Figure S3: Protein-ligand contacts in simulation timeline. The synthesis of the studied agents and the physicochemical characteristics of the compounds are also given in the SM.
