# Supplementary material for: Borneol Ester Derivatives as Entry Inhibitors of a Wide Spectrum of SARS-CoV-2 Viruses
Source: Viruses. 2022 Jun 14;14(6):1295. doi: 10.3390/v14061295 (PMC9228966; doi:10.3390/v14061295)
Supplement: Supplementary file 1 [file viruses-14-01295-s001.zip › viruses-1708889-supplementary.pdf]

Borneol ester derivatives as entry inhibitors of a wide spectrum of SARS-CoV-2 viruses

Olga I. Yarovaya [1,*], Dmitriy N. Shcherbakov [2], Sophia S. Borisevich [3], Anastasiya S. Sokolova [1], Maxim A. Gureev [4,5], Edward M. Khamitov [3], Nadezda B. Rudometova [2], Anastasiya V. Zybkina [2], Ekaterina D. Mordvinova [1,2], Anna V. Zaykovskaya [2], Artem D. Rogachev [1], Oleg V. Pyankov [2], Rinat A. Maksyutov [2] and Nariman F. Salakhutdinov [1]

1 Department of Medicinal Chemistry, N.N. Vorozhtsov Novosibirsk Institute of Organic Chemistry SB RAS, Lavrentiev ave., 9, 630090 Novosibirsk, Russia; asokolova@nioch.nsc.ru (A.S.S.); mordvinova97@mail.ru (E.D.M.); artrogachev@yandex.ru (A.D.R.); anvar@nioch.nsc.ru (N.F.S.)
2 State Research Center of Virology and Biotechnology VECTOR, Rospotrebnadzor, 630559 Koltsovo, Russia; dnshcherbakov@gmail.com (D.N.S.); andreeva_nb@vector.nsc.ru (N.B.R.); zybkina_av@vector.nsc.ru (A.V.Z.); zaykovskaya_av@vector.nsc.ru (A.V.Z.); pyankov_ov@vector.nsc.ru (O.V.P.); maksyutov_ra@vector.nsc.ru (R.A.M.)
3 Laboratory of Chemical Physics Ufa Institute of Chemistry, Ufa Federal Research Center, RAS, Octyabrya pr., 71, 450054 Ufa, Russia; monrel@mail.ru (S.S.B.); khamitovem@gmail.com (E.M.K.)
4 Research Center "Digital Biodesign and Personalized Healthcare", I.M. Sechenov First Moscow State Medical University, Trubetskaya str., 8/2, 119991 Moscow, Russia; max_technik@mail.ru
5 Department of Computational Biology, Sirius University of Science and Technology, Olympic Ave., 1, 354340 Sochi, Russia
* Correspondence: ooo@nioch.nsc.ru

Supplementary information

Molecular Modeling Study

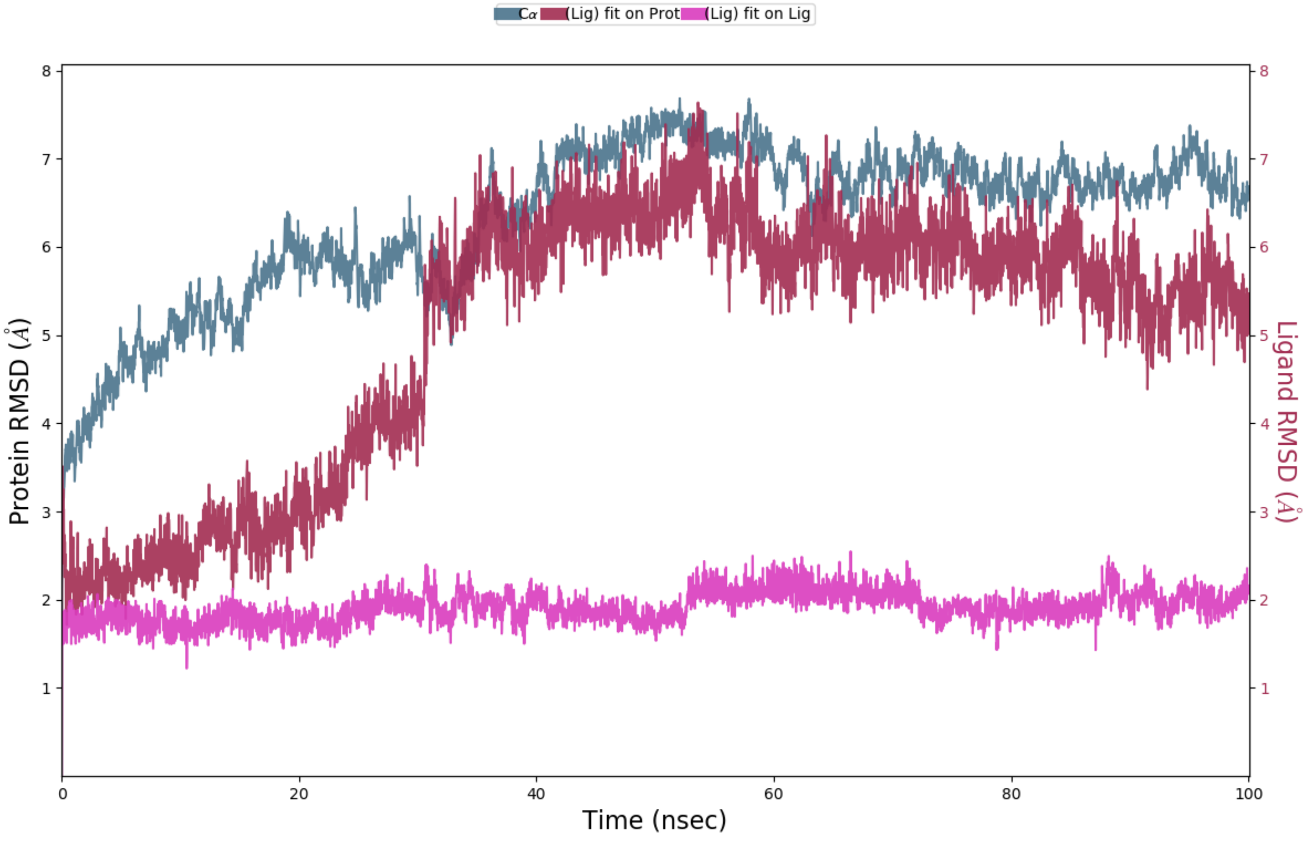

Figure S1: System RMSD during simulation.

The Root Mean Square Deviation (RMSD) is used to measure the average change in displacement of a selection of atoms for a particular frame with respect to a reference frame. It is calculated for all frames in the trajectory. The RMSD for frame x is: where N is the number of atoms in the atom selection; tref is the reference time, (typically the first frame is

used as the reference and it is regarded as time t=0); and r' is the position of the selected atoms in frame x after superimposing on the reference frame, where frame x is recorded at time tx. The procedure is repeated for every frame in the simulation trajectory.

Protein RMSD: The above plot shows the RMSD evolution of a protein (left Y-axis). All protein frames are first aligned on the reference frame backbone, and then the RMSD is calculated based on the atom selection. Monitoring the RMSD of the protein can give insights into its structural conformation throughout the simulation. RMSD analysis can indicate if the simulation has equilibrated — its fluctuations towards the end of the simulation are around some thermal average structure. Changes of the order of 1-3 Å are perfectly acceptable for small, globular proteins. Changes much larger than that, however, indicate that the protein is undergoing a large conformational change during the simulation. It is also important that your simulation converges — the RMSD values stabilize around a fixed value. If the RMSD of the protein is still increasing or decreasing on average at the end of the simulation, then your system has not equilibrated, and your simulation may not be long enough for rigorous analysis.

Ligand RMSD: Ligand RMSD (right Y-axis) indicates how stable the ligand is with respect to the protein and its binding pocket. In the above plot, 'Lig fit Prot' shows the RMSD of a ligand when the protein-ligand complex is first aligned on the protein backbone of the reference and then the RMSD of the ligand heavy atoms is measured. If the values observed are significantly larger than the RMSD of the protein, then it is likely that the ligand has diffused away from its initial binding site. 'Lig fit Lig' shows the RMSD of a ligand that is aligned and measured just on its reference conformation. This RMSD value measures the internal fluctuations of the ligand atoms.

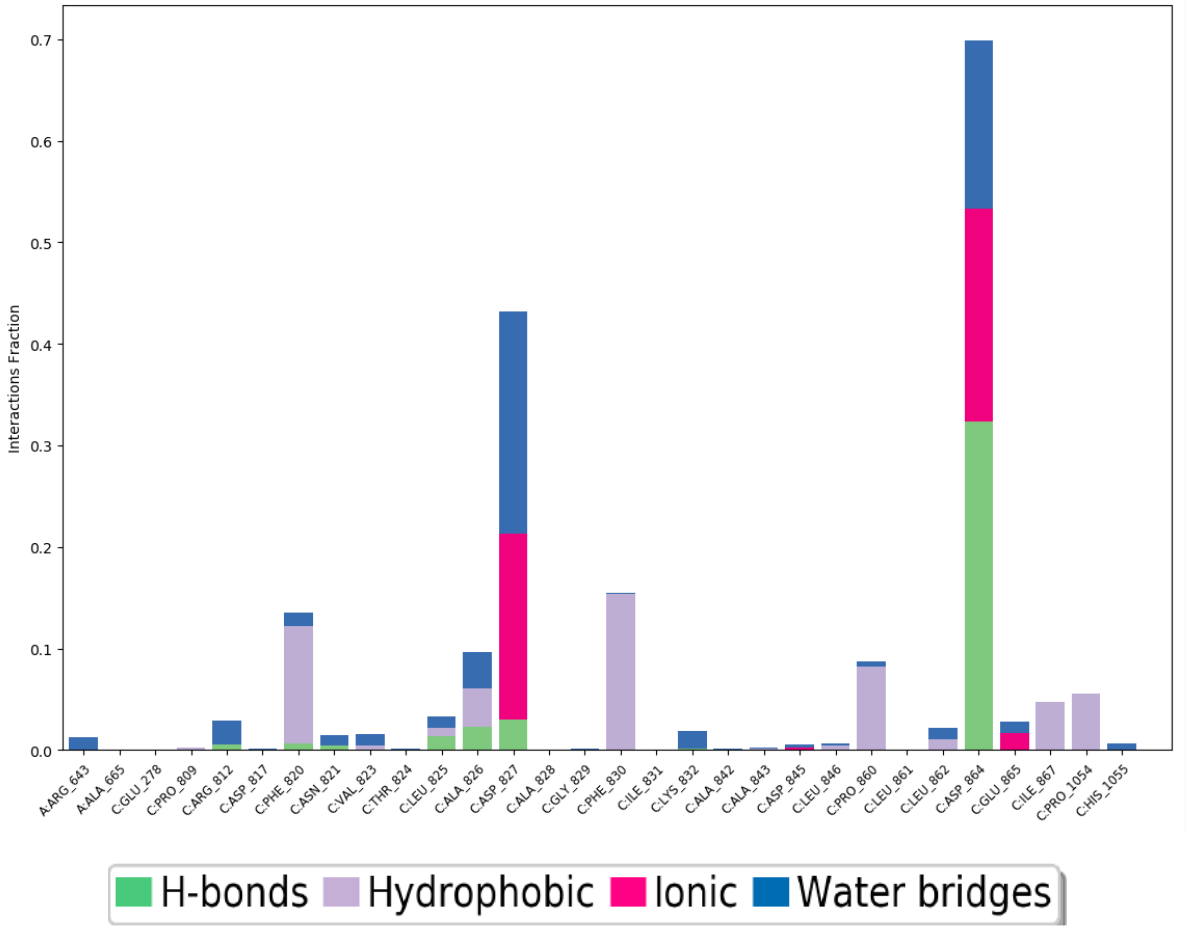

Figure S2: Ligand-protein interactions percentage and type

Protein interactions with the ligand can be monitored throughout the simulation. These interactions can be categorized by type and summarized, as shown in the plot above. Protein-ligand interactions (or 'contacts') are categorized into four types: Hydrogen Bonds, Hydrophobic, Ionic and Water Bridges. Each interaction type contains more specific subtypes. The stacked bar charts are normalized over the course of the trajectory: for example, a value of 0.7 suggests that 70% of the simulation time the specific interaction is maintained. Values over 1.0 are possible as some protein residue may make multiple contacts of same subtype with the ligand.

Hydrogen Bonds: (H-bonds) play a significant role in ligand binding. Consideration of hydrogen-bonding properties in drug design is important because of their strong influence on drug specificity, metabolization and adsorption. Hydrogen bonds between a protein and a ligand can be further broken down into four subtypes: backbone acceptor; backbone donor; side-chain acceptor; side-chain donor. The current geometric criteria for protein-ligand H-bond is: distance of 2.5 Å between the donor and acceptor atoms (D—H···A); a donor angle of ≥ 120° between the donor-hydrogen-acceptor atoms (D—H···A); and an acceptor angle of ≥ 90° between the hydrogen-acceptor-bonded_atom atoms (H···A—X).

Hydrophobic contacts: fall into three subtypes: π-Cation; π-π; and Other, non-specific interactions. Generally, these types of interactions involve a hydrophobic amino acid and an aromatic or aliphatic group on the ligand, but we have extended this category to also include π-Cation interactions. The current geometric criterion for hydrophobic interactions is as follows: π-Cation — Aromatic and charged groups within 4.5 Å; π-π — Two aromatic groups stacked face-to-face or face-to-edge; Other — A non-specific hydrophobic sidechain within 3.6 Å of a ligand's aromatic or aliphatic carbons.

Ionic interactions: or polar interactions, are between two oppositely charged atoms that are within 3.7 Å of each other and do not involve a hydrogen bond. We also monitor Protein-Metal-Ligand interactions, which are defined by a metal ion coordinated within 3.4 Å of protein's and ligand's heavy atoms (except carbon). All ionic interactions are broken down into two subtypes: those mediated by a protein backbone or side chains.

Water Bridges: are hydrogen-bonded protein-ligand interactions mediated by a water molecule. The hydrogen-bond geometry is slightly relaxed from the standard H-bond definition. The current geometric criteria for a protein-water or water-ligand H-bond are: a distance of 2.8 Å between the donor and acceptor atoms (D—H···A); a donor angle of ≥110° between the donor-hydrogen-acceptor atoms (D—H···A); and an acceptor angle of ≥90° between the hydrogen-acceptor-bonded_atom atoms (H···A—X).

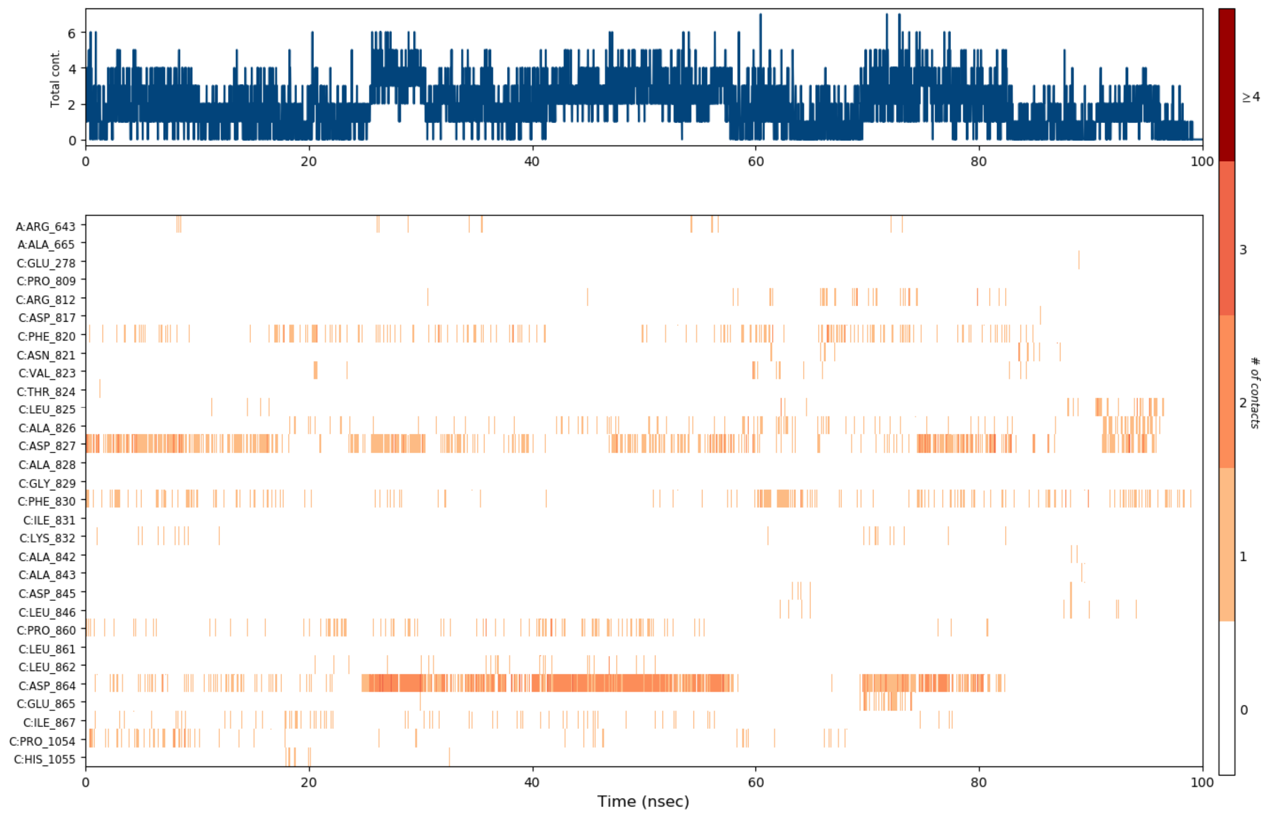

Figure S3 – Protein-ligand contacts in simulation timeline

A timeline representation of the interactions and contacts (H-bonds, Hydrophobic, Ionic, Water bridges) summarized in the figure SM-3. The top panel shows the total number of specific contacts the protein makes with the ligand over the course of the trajectory. The bottom panel shows which residues interact with the ligand in each trajectory frame. Some residues make more than one specific contact with the ligand, which is represented by a darker shade of orange, according to the scale to the right of the plot.

Chemistry

Scheme for the synthesis of camphor imines

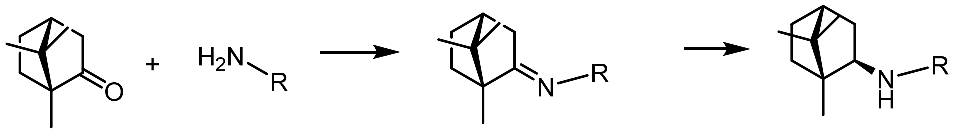

(+)-Camphor **1, 3-5** **2**

2-(1,7,7-Trimethylbicyclo[2.2.1]heptan-2-ylideneamino) ethanol (**1**). A mixture of (1R)-camphor (1.0 equiv.), aminoethanol (2.5 equiv.), and anhydrous $ZnCl_2$ (0.1% mol on camphor) was reflux for 12 h. Diethyl ether was added to the reaction mixture, after completion of the reaction. The organic layer was washed with brine, dried (Na_2SO_4) and evaporated. The crude product purified by vacuum distillation (bp 110 °C at 5 mm Hg) to give white solid (yield 85%). NMR ^{1}H (600MHz, $CDCl_3$, δ, ppm, J/Hz): 0.71 (3H, s, Me-9), 0.90 (3H, s, Me-10), 0.93 (3H, s, Me-8), 1.17 (1H, ddd, ^{2}J=12.4, $J_{4endo, 5endo}$=9.4, $J_{4endo, 5exo}$=4.2, H-4*endo*), 1.31 (1H, ddd, ^{2}J=13.1, $J_{5endo, 4endo}$=9.4, $J_{5endo, 4exo}$=4.5, H-5*endo*), 1.64 (1H, dddd, ^{2}J=13.1, $J_{5exo, 4exo}$=11.5, $J_{5exo, 4endo}$=4.2, $J_{5exo, 2exo}$=0.5, H-5*exo*), 1.80 (1H, d, ^{2}J=16.8, H-2*endo*), 1.82 (1H, ddddd, ^{2}J=12.4, $J_{4exo, 5exo}$=11.5, $J_{4exo, 5endo}$=4.5, $J_{4exo, 3}$= 4.4, $J_{4exo, 2exo}$=3.1, H-4*exo*), 1.92 (1H, dd, $J_{3, 2exo}$=4.6, $J_{3, 4exo}$=4.4, H-3), 2.31 (1H, dddd, ^{2}J=16.8, $J_{2exo, 3}$=4.6, $J_{2exo, 4exo}$=3.1, $J_{2exo, 5exo}$=0.5, H-2*exo*), 2.71 (1H, br.s, O<u>H</u>), 3.20-3.33 (2H,

m, H-11), 3.72-3.81 (2H, m, H-12). NMR ^{13}C (125 МГц, $CDCl_3$, δ, ppm): δ 184.25 s (C-1), 62.04 t (C-12), 53.66 s (C-6), 53.62 t (C-11), 46.96 s (C-7), 43.67 d (C-3), 35.85 t (C-2), 31.99 t (C-5), 27.23 t (C-4), 19.37 q (Me-9), 18.75 (Me-10), 11.01 (Me-8). $[\alpha]_D^{31}$=-25.0 ($CHCl_3$, c=0.96). HRMS: *m/z* calcd. for $C_{12}H_{21}ON$: 195.1618. Found: 195.1616. Mp=64ºC.

2-(1,7,7-Trimethylbicyclo[2.2.1]heptan-2-ylamino)ethanol (**2**). Corresponding camphor imine **1** (5 mmol) and $NiCl_2{\cdot}6H_2O$ (10 mmol) were taken in methanol (50 ml) and cooled to –30°C. $NaBH_4$ (50 mmol) was added in portions to this mixture under stirring at -30°C over a period of 30 min. The reaction mixture was further stirred for 1h at -30°C and for 4h at room temperature. 25% NaOH solution (7 ml) and ether (50 ml) were added, and the contents were filtered. The organic layer was washed with saturated NaCl solution (2 x 10 ml), dried over anhydrous sodium sulfate. The solvent was removed under reduced pressure and crude product purified by flash silica gel column chromatography. NMR ^1H (400MHz, $CDCl_3$, δ, ppm, J/Hz): 0.78 (3H, s, Me-9), 0.85 (3H, s, Me-8), 0.96 (3H, s, Me-10), 0.98-1.08 (2H, m, H-4*endo*,5*endo*), 1.43-1.72 (5H, m, H-2*exo*,2*endo*,3,4*exo*,5*exo*), 2.50 (1H, dd, $J_{1endo,\ 2endo}$=8.0, $J_{1endo,\ 2exo}$=5.2, H-1*endo*), 2.61-2.74 (2H, m, H-11) 3.51-3.55 (2H, t-like m, H-12) NMR ^{13}C (75 MHz, $CDCl_3$, δ, ppm): 66.36 d (C-1), 60.93 t (C-12), 49.76 t (C-11), 48.32 s (C-6), 46.52 s (C-7), 45.02 d (C-3), 39.15 t (C-2), 36.84 t (C-5), 27.14 t (C-4), 20.47 q and 20.40 q (Me-9 and Me-10), 12.09 q (Me-8). $[\alpha]_D^{25}$ =72.0 ($CHCl_3$, c=1.0). HRMS: *m/z* calcd. for $C_{12}H_{23}ON$: 197.1774 . Found: 197.1778.

(E)-N^1,N^1-Diethyl-N^2-((1R,4R)-1,7,7-trimethylbicyclo[2.2.1]heptan-2-ylidene)ethane-1,2-diamine (**3**). (+)-Camphor (26 mmol) and N^1,N^1-diethylethane-1,2-diamine (30 mmol) were mixed in toluene with catalytic BF_3Et_2O (1– 5 mol %), followed by azeotropic removal water with Dean–Stark for 15 h. Then the reaction mixture was washed with saturated NaCl solution. The organic layer was separated. The aqueous layer was extracted with CH_2Cl_2 (10 ml). The combined organic phases were dried over Na_2SO_4. The solvent was evaporated. The residue was separated by vacuum distillation. T = 100–102 °C at 1 torr. As a result we have obtained **3** in 94% yield (4.7 g, 24.4 mmol). $[\alpha]_D^{25}$=18.7 (c 1.1, $CHCl_3$) ^1H NMR (500 MHz, CDCl3, d, ppm, J/Hz): 0.65 (3H, s, Me-9), 0.81 (3H, s, Me-10), 0.84 (3H, s, Me-8), 0.92 (6H, t, $J_{14,\ 13}$=7.1, Me-14 and Me-16), 1.08 (1H, ddd, ^2J=12.3, $J_{4endo,\ 5endo}$=9.3, $J_{4endo,\ 5exo}$=4.2, H-4_{endo}), 1.24 (1H, ddd, ^2J=12.3, $J_{5endo,\ 4endo}$=9.3, $J_{5endo,\ 4exo}$=4.5, H-5_{endo}), 1.54 (1H, ddd, ^2J=$J_{5exo,\ 4exo}$=12.3, $J_{5exo,\ 4endo}$=4.2, H-5_{exo}), 1.73 (1H, d, ^2J=16.9, H-2_{endo}), 1.73 (1H, ddddd, ^2J=$J_{4exo,\ 5exo}$=12.3, $J_{4exo,\ 5endo}$=$J_{4exo,\ 3}$= 4.5, $J_{4exo,\ 2exo}$=3.2, H-4_{exo}), 1.81 (1H, dd, $J_{3,\ 2exo}$=$J_{3,\ 4exo}$=4.5, H-3), 2.24 (1H, ddd, ^2J=16.9, $J_{2exo,\ 3}$=4.5, $J_{2exo,\ 4exo}$=3.2, H-2_{exo}), 2.45 and 2.45 (each 2H, q, $J_{13,\ 14}$=7.1, H-13 and H-15), 2.55 (2H, t, $J_{12,\ 11}$=7.6, H-12), 3.18 and 3.25 (each 1H, dt, ^2J=12.1, $J_{11,\ 12}$=7.6, H-11). NMR ^{13}C (125 MHz, $CDCl_3$, δ, ppm): 181.90 s (C-1), 53.22 t (C-12), 53.20 c (C-6), 50.73 t (C-11), 47.22 t (C-13 и C-15), 46.64 s (C-7), 43.61 d (C-3), 35.26 t (C-2), 31.89 t (C-5), 27.22 t (C-4), 19.29 q (C-9), 18.70 q (C-10), 11.71 q (C-14 and C-16), 11.14 q (C-8). HR-MS: 250.2402 (M^+ $C_{16}H_{30}N_2$; calcd 250.2404)

3-Morpholino-N-((1R,4R)-1,7,7-trimethylbicyclo[2.2.1]heptan-2-ylidene)propan-1-amine (**4**). A mixture of (1*R*)-(+)-camphor (1.0 equiv.), 3-morpholinopropan-1-amine (2.5 equiv.), and anhydrous $ZnCl_2$ (0.1 % mol on camphor) was reflux for 12 h. Diethyl ether was added to the reaction mixture, after completion of the reaction. The organic layer was washed with brine, dried (Na_2SO_4) and evaporated. The crude product was purified by flash silica gel column chromatography (hexane-ethyl acetate eluent). NMR ^1H (400MHz, $CDCl_3$, δ, ppm, J/Hz): 0.70 (3H, s, Me-9), 0.88 (3H, s, Me-10), 0.91 (3H, s, Me-8), 1.14 (1H, ddd, ^2J=12.4, $J_{4endo,\ 5endo}$=9.4, $J_{4endo,}$

$_{5exo}$=4.2, H-4*endo*), 1.29 (1H, ddd, ^2J=13.1, $J_{5endo, 4endo}$=9.4, $J_{5endo, 4exo}$=4.5, H-5*endo*), 1.61 (1H, dddd, ^2J=13.1, $J_{5exo, 4exo}$=11.5, $J_{5exo, 4endo}$=4.2, $J_{5exo, 2exo}$=0.6, H-5*exo*), 1.70-1.78 (2H, m, H-12), 1.80 (1H, ddddd, ^2J=12.4, $J_{4exo, 5exo}$=11.5, $J_{4exo, 5endo}$=4.5, $J_{4exo, 3}$= 4.4, $J_{4exo, 2exo}$=3.1, H-4*exo*), 1.81 (1H, d, ^2J=16.8, H-2*endo*), 1.89 (1H, dd, $J_{3, 2exo}$=4.6, $J_{3, 4exo}$=4.4, H-3), 2.27-2.34 (3H, m, H-2*exo*,H-13), 2.35-2.41 (4H, m, H-14,17), 3.14-3.27 (2H, m, H-11), 3.65-3.68 (4H, m, H-15,16). NMR ^{13}C (125 MHz, $CDCl_3$, δ, ppm): 181.68 s (C-1), 66.89 t (C-15,16), 56.69 t (C-13), 53.62 t (C-14,17), 53.36 s (C-6), 49.98 t (C-11), 46.72 s (C-7), 43.73 d (C-3), 35.28 t (C-2), 32.10 t (C-5), 27.38 t (C-4), 27.26 t (C-12), 19.40 q (Me-9), 18.82 q (Me-10), 11.29 q (Me-8). $[\alpha]_D^{27}$=18.0 ($CHCl_3$, c=0.9). HRMS: *m/z* calcd. for $C_{17}H_{30}ON_2$: 278.2353. Found: 278.2350.

2-(3,4-dimethoxyphenyl)-N-(1,7,7-trimethylbicyclo[2.2.1]heptan-2-ylidene)ethanamine (**5**). Compound **5** was prepared similar to synthesis compound **4**. NMR ^1H (500MHz, $CDCl_3$, δ, ppm, J/Hz): 0.57 (3H, s, Me-9), 0.84 (3H, s, Me-10), 0.92 (3H, s, Me-8), 0.99 (1H, ddd, ^2J=12.3, $J_{4endo, 5endo}$=9.3, $J_{4endo, 5exo}$=4.2, H-4*endo*), 1.23 (1H, ddd, ^2J=12.3, $J_{5endo, 4endo}$=9.3, $J_{5endo, 4exo}$=4.5, H-5*endo*), 1.57 (1H, d, ^2J=16.9, H-2*endo*), 1.58 (1H, ddd, ^2J=$J_{5exo, 4exo}$=12.3, $J_{5exo, 4endo}$=4.2 , H-5*exo*), 1.73 (1H, ddddd, ^2J=$J_{4exo, 5exo}$=12.3, $J_{4exo, 5endo}$=$J_{4exo, 3}$= 4.5, $J_{4exo, 2exo}$=3.2, H-4*exo*), 1.79 (1H, dd, $J_{3, 2exo}$=$J_{3, 4exo}$=4.5, H-3), 2.14 (1H, ddd, ^2J=16.9, $J_{2exo, 3}$=4.5, $J_{2exo, 4exo}$=3.2, H-2*exo*), 2.77-2.87 (2H, m, H-12), 3.38 and 3.45 (each 1H, dt, ^2J=12.0, $J_{11, 12}$=7.2, H-11), 3.79 (3H, s, O<u>Me</u>-19), 3.81 (3H, s, O<u>Me</u>-20), 6.68-6.78 (3H, m, H-14, H-17, H-18). NMR ^{13}C (125 MHz, $CDCl_3$, δ, ppm): 182.01 s (C-1), 148.50 s (C-16), 147.15 s (C-15), 133.33 s (C-13), 120.68 d (C-18), 112.46 d (C-14), 111.15 d (C-17), 55.82 q (C-19), 55.61 q (C-20), 54.04 t (C-11), 53.33 s (C-6), 46.60 s (C-7), 43.59 d (C-3), 36.44 t (C-12), 35.26 t (C-2), 32.04 t (C-5), 27.21 t (C-4), 19.21 q (Me-9), 18.74 q (Me-10), 11.32 q (Me-8). $[\alpha]_D^{27}$=24.4 ($CHCl_3$, c=1.6). HRMS: *m/z* calcd. for $C_{20}H_{29}O_2N$: 315.2193. Found: 315.2186.

Synthesis of (-)-borneol esters

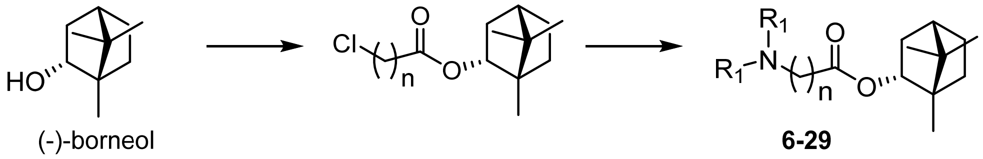

(-)-borneol **6-29**

(1S,2R,4S)-1,7,7-Trimethylbicyclo[2.2.1]heptan-2-yl 2-chloroacetate. To the mixture of (−)-borneol (0.03 mol) and Et_3N (0.03 mol) in 20 ml dry CH_2Cl_2 at 15–18 °C in an Ar atmosphere was added chloroacetyl chloride (0.05 mol), and the mixture was stirred at 23–25 °C for 12 h. The organic layers were washed with brine and extracted with CH_2Cl_2. The combined organic phase was dried over anhydrous Na_2SO_4 and the solvent was removed. The crude product was purified by vacuum distillation (bp 105 °C at 5 mm Hg). Yield: 65%, colourless oil.

(1S,2R,4S)-1,7,7-Trimethylbicyclo[2.2.1]heptan-2-yl 3-chloropropanoate. To the solution of 3-chloropropanoic acid in dry CH_2Cl_2, excess oxalyl chloride and N,N-dimethylformamide (one drop) were added. The mixture was stirred at room temperature for 4 h in an Ar atmosphere. The excess oxalyl chloride was removed on a rotary evaporator. The resulting chloroanhydride of 3-chloropropanoic acid was used in a further reaction immediately. To the solution of (−)-borneol (30 mmol) in CH_2Cl_2 was added the chloroanhydride (30 mmol) in CH_2Cl_2 (5 ml) and Et_3N (30 mmol) at 0–5 °C, and the mixture was stirred at room temperature for 24 h in an atmosphere of Ar. The resulting precipitate was filtrated; the filtrate was

washed with brine and extracted with CH_2Cl_2. The combined organic phase was dried over anhydrous Na_2SO_4 and the solvent was removed. The crude product was purified by flash CC (silica gel, eluent: hexane–ethyl acetate). Yield: 59%, yellow oil.

(1S,2R,4S)-1,7,7-Trimethylbicyclo[2.2.1]heptan-2-yl 4-chlorobutanoate and *(1S,2R,4S)-1,7,7-trimethylbicyclo[2.2.1]heptan-2-yl 5-chloropentanoate* were synthesized as described above.

General synthetic procedure for compounds 6-18, 21

A mixture of 1,7,7-trimethylbicyclo[2.2.1]heptan-2-yl 2-chloroacetate or 1,7,7-trimethylbicyclo[2.2.1]heptan-2-yl 3-chloropropanoate (2 mmol), the corresponding amine (2.2 mmol), Et_3N (2.2 mmol) and 15 ml MeOH were stirred at room temperature for 24 h. After completion, the mixture was concentrated in a vacuum. Brine and ethyl acetate were added to the residue, and then the mixture was extracted twice with ethyl acetate. The combined organic phase was dried over Na_2SO_4 and the solvent was removed in vacuo. The crude product was purified by silica gel CC (eluent: hexane–ethyl acetate).

(1S,2R,4S)-1,7,7-Trimethylbicyclo[2.2.1]heptan-2-yl 2-morpholinoacetate (**6**). Yield: 62%, yellow oil. ^{1}H NMR (δ, ppm, J/Hz): 0.79 (3H, s, Me-9), 0.84 (3H, s, Me-10), 0.87 (3H, s, Me-8), 0.93 (1H, dd, ^{2}J=13.7, $J_{2endo, 1exo}$=3.5, H-2endo), 1.13-1.31 (2H, m, H-4endo, H-5exo), 1.64 (1H, dd, $J_{3, 2exo}$=$J_{3, 4exo}$=4.6, H-3), 1.66-1.76 (1H, m, H-4exo), 1.86 (1H, ddd, ^{2}J=12.9, $J_{5endo, 4endo}$=9.3, $J_{5endo, 4exo}$=4.4, H-5endo), 2.33 (1H, m, H-2exo), 2.55-2.62 (4H, m, 2H-13, 2H-16), 3.21 (2H, s, H-12), 3.69-3.75 (4H, m, 2H-14, 2H-15), 4.91 (1H, ddd, $J_{1exo,2exo}$=10.0, $J_{1exo, 2endo}$=3.5, $J_{1exo, 5exo}$=2.2, H-1exo). ^{13}C NMR (δ, ppm): 170.25 s (C-11), 70.10 d (C-1), 66.64 t (C-14, C-15), 59.53 t (C-12), 53.04 t (C-13, C-16), 48.56 s (C-6), 47.61 s (C-7), 44.62 d (C-3), 36.58 t (C-2), 27.80 t (C-4), 26.91 t (C-5), 19.49 q (Me-9), 18.62 q (Me-10), 13.35 q (Me-8). $[\alpha]_D^{26}$ =-30.6 ($CHCl_3$, c=1.2). HRMS: calcd for $C_{16}H_{27}O_3N$: 281.1986; found: 281.1991.

(1S,2R,4S)-1,7,7-Trimethylbicyclo[2.2.1]heptan-2-yl 2-(4-methylpiperazin-1-yl)acetate (**7**). Yield: 60%, pale yellow oil. ^{1}H NMR (δ, ppm, J/Hz): 0.77 (3H, s, Me-10), 0.82 (3H, s, Me-8), 0.85 (3H, s, Me-9), 0.94 (1H, dd, ^{2}J=13.7, $J_{2endo, 1exo}$=3.5, H-2endo), 1.12-1.29 (2H, m, H-4endo, H-5exo), 1.64 (1H, dd, $J_{3, 2exo}$=$J_{3, 4exo}$=4.6, H-3), 1.64-1.75 (1H, m, H-4exo), 1.86 (1H, ddd, ^{2}J=12.9, $J_{5endo, 4endo}$=9.3, $J_{5endo, 4exo}$=4.4, H-5endo), 2.24 (3H, s, Me-15), 2.26-2.36 (1H, m, H-2exo), 2.36-2.66 (8H, br. s., 2H-13, 2H-14, 2H-16, 2H-17), 3.17 (2H, s, H-12), 4.89 (1H, ddd, $J_{1exo, 2exo}$=10.0, $J_{1exo, 2endo}$=3.5, $J_{1exo,5exo}$=2.2, H-1exo). ^{13}C NMR (δ, ppm): 170.45 s (C-11), 80.07 d (C-1), 59.32 t (C-12), 54.73 t (C-13, C-17), 52.82 (C-14, C-16), 48.63 s (C-6), 47.80 s (C-7), 45.86 k (Me-15), 44.70 d (C-3), 36.63 t (C-2), 27.87 t (C-4), 26.95 t (C-5), 19.56 q (Me-9), 18.69 q (Me-10), 13.41 q (Me-8). $[\alpha]_D^{25}$ =-31.9 ($CHCl_3$, c=1.18). HRMS: calcd for $C_{17}H_{30}O_2N_2$: 294.2302; found: 294.2303.

(1S,2S,4S)-1,7,7-Trimethylbicyclo[2.2.1]heptan-2-yl 2-(4-(2-hydroxyethyl)piperazin-1-yl)acetate (**8**). Yield 36%; pale yellow oil. ^{1}H NMR (δ, ppm, J/Hz): 0.76 (3H, s, Me-10), 0.80 (3H, s, Me-8), 0.83 (3H, s, Me-9), 0.91 (1H, dd, ^{2}J=14.2, $J_{2endo, 1exo}$=3.2, H-2endo), 1.11-1.28 (2H, m, H-4endo, H-5exo), 1.59-1.73 (2H, m, H-4exo, H-3), 1.78-1.88 (1H, m, H-5endo), 2.23-2.35 (1H, m, H-2exo), 2.43-2.67 (10H, br. s., H-13, H-14, H-15, H-16, H-17), 2.94 (1H, br. s., OH), 3.17 (2H, s, H-12), 3.54 (2H, t, J=5.4, H-18), 4.87 (1H, m, H-1exo). ^{13}C NMR (δ, ppm): 170.34 s (C-11), 79.96 d (C-1), 59.09 t (C-18), 59.05 t (C-12), 57.47 t (C-17), 52.62 and 52.45 t (C-13, C-14, C-15, C-16),

48.50 s (C-6), 47.54 s (C-7), 44.55 d (C-3), 36.52 t (C-2), 27.75 t (C-4), 26.84 t (C-5), 19.45 q (Me-9), 18.58 q (Me-8), 13.32 q (Me-10). HR-MS: 324.2413 (M^+, $C_{18}H_{32}O_3N_2$; calcd 324.2407).

(1S,2R,4S)-1,7,7-trimethylbicyclo[2.2.1]heptan-2-yl 2-(4-(2-aminoethyl)piperazin-1-yl)acetate (**9**). Yield 25%; brown oil. ^{1}H NMR (δ, ppm, J/Hz): 0.76 (3H, s, Me-10), 0.80 (3H, s, Me-8), 0.84 (3H, s, Me-9), 0.91 (1H, dd, ^2J=14.2, $J_{2endo, 1exo}$=3.2, H-2endo), 1.11-1.29 (2H, m, H-4endo, H-5exo), 1.59-1.73 (2H, m, H-4exo, H-3), 1.80-1.88 (1H, m, H-5endo), 2.08 (2H, br. s., NH_2), 2.25-2.34 (1H, m, H-2exo), 2.37 (2H, t, J=6.0, H-17), 2.39-2.62 (8H, br. s., H-13, H-14, H-15, H-16), 2.72 (2H, t, J=6.0, H-18), 3.17 (2H, s, H-12), 4.87 (1H, m, H-1exo). ^{13}C NMR (δ, ppm): 170.55 s (C-11), 80.07 d (C-1), 60.92 t (C-17), 59.36 t (C-12), 52.91 and 52.88 t (C-13, C-14, C-15, C-16), 48.65 s (C-6), 47.69 s (C-7), 44.72 d (C-3), 38.66 t (C-18), 36.66 t (C-2), 27.89 t (C-4), 26.98 t (C-5), 19.58 q (Me-9), 18.71 q (Me-8), 13.44 q (Me-10). HR-MS: 323.2554 (M^+, $C_{18}H_{33}O_2N_3$; calcd 323.2567).

(1S,2R,4S)-1,7,7-trimethylbicyclo[2.2.1]heptan-2-yl 2-(4-benzylpiperazin-1-yl)acetate (***10***). Yield 42%; yellow oil. ^{1}H NMR (δ, ppm, J/Hz): 0.80 (3H, s, Me-10), 0.84 (3H, s, Me-8), 0.87 (3H, s, Me-9), 0.95 (1H, dd, ^2J=13.7, $J_{2endo, 1exo}$=3.3, H-2endo), 1.14-1.31 (2H, m, H-4endo, H-5exo), 1.63-1.66 (1H,m, H-3), 1.68-1.76 (1H, m, H-4exo), 1.83-1.92 (1H, m, H-5endo), 2.27-2.37 (1H, m, H-2exo), 2.46-2.64 (8H, br. s., H-13, H-14, H-15, H-16), 3.20 (2H, s, H-12), 3.50 (2H, s, H-17), 4.91 (1H, m, H-1exo), 7.19-7.24 (1H, m, H-21), 7.27-7.30 (4H, m, H-19, H-20, H-22, H-23). ^{13}C NMR (δ, ppm): 170.56 s (C-11), 138.00 s (C-18), 129.04 d (C-19, C-23), 128.06 d (C-20, C-22), 126.88 d (C-21), 80.02 d (C-1), 62.83 t (C-17), 59.36 t (C-12), 52.86 and 52.73 t (C-13, C-14, C-15, C-16), 48.63 s (C-6), 47.67 s (C-7), 44.69 d (C-3), 36.64 t (C-2), 27.87 t (C-4), 26.96 t (C-5), 19.57 q (Me-9), 18.71 q (Me-8), 13.43 q (Me-10). HR-MS: 370.2614 (M^+, $C_{23}H_{34}O_2N_2$; calcd 370.2615).

(1S,2R,4S)-1,7,7-trimethylbicyclo[2.2.1]heptan-2-yl 2-(dibutylamino)acetate (**11**). Yield: 75%, pale yellow oil. ^{1}H NMR (δ, ppm, J/Hz): 0.80 (3H, s, Me-9), 0.84 (3H, s, Me-10), 0.87 (3H, s, Me-8), 0.88 (6H, t, J=7.26, Me-19, Me-20), 0.94 (1H, dd, ^2J=13.8, $J_{2endo, 1exo}$=3.5, H-2endo), 1.15-1.34 (6H, m, H-4endo, H-5exo, 2H-17, 2H-18), 1.37-1.46 (4H, m, 2H-15, 2H-16), 1.64 (1H, m, H-3), 1.67-1.77 (1H, m, H-4exo), 1.92 (1H, m, H-5endo), 2.33 (1H, m, H-2exo), 2.51-2.58 (4H, m, 2H-13, 2H-14), 3.31 (2H, s, H-12), 4.90 (1H, ddd, $J_{1exo, 2exo}$=10.0, $J_{1exo,2endo}$=3.5, $J_{1exo, 5exo}$=2.2, H-1exo). ^{13}C NMR (δ, ppm): 172.00 s (C-11), 79.75 d (C-1), 54.95 t (C-12), 54.03 (C-13, C-14), 48.55 s (C-6), 47.62 s (C-7), 44.72 d (C-3), 36.73 t (C-2), 29.72 t (C-15, C-16), 27.88 t (C-4), 27.07 t (C-5), 20.40 t (C-17, C-18), 19.56 q (Me-9), 18.68 q (Me-10), 13.91 q (Me-19, Me-20), 13.38 q (Me-8). $[\alpha]_D^{26}$ =-30 ($CHCl_3$, c=1.28). HRMS: calcd for $C_{20}H_{37}O_2N$: 323.2819; found: 323.2823.

(1S,2R,4S)-1,7,7-trimethylbicyclo[2.2.1]heptan-2-yl 3-morpholinopropanoate (**12**). Yield: 58%, pale yellow oil. ^{1}H NMR (δ, ppm, J/Hz): 0.80 (3H, s, Me-9), 0.83 (3H, s, Me-10), 0.87 (3H, s, Me-8), 0.94 (1H, dd, ^2J=13.7, $J_{2endo, 1exo}$=3.5, H-2endo), 1.13-1.30 (2H, m, H-4endo, H-5exo), 1.65 (1H, dd, $J_{3, 2exo}$=$J_{3, 4exo}$=4.6, H-3), 1.71 (1H, ddddd, ^2J=$J_{4exo, 5exo}$=12.0, $J_{4exo,3}$=4.6, $J_{4exo, 5endo}$=4.4, $J_{4exo, 2exo}$=3.3, H-4exo), 1.88 (1H, ddd, ^2J=12.9, $J_{5endo, 4endo}$=9.3, $J_{5endo,4exo}$=4.4, H-5endo), 2.31 (1H, m, H-2exo), 2.38-2.45 (4H, m, 2H-14, 2H-17), 2.45-2.49 (2H, m, H-12), 2.62-2.68 (2H, m, H-13), 3.60-3.73 (4H, m, 2H-15, 2H-16), 4.87 (1H, ddd, $J_{1exo,2exo}$=10.0, $J_{1exo,2endo}$=3.5, $J_{1exo,5exo}$=2.2, H-1exo). ^{13}C NMR (δ, ppm): 172.56 s (C-11), 79.76 d (C-1), 66.83 t (C-15, C-16), 54.06 t (C-13), 53.26 t (C-14, C-17), 48.69 s (C-6), 47.68 s (C-7), 44.75 d (C-3), 36.55 t (C-2), 32.50 t (C-12), 27.94 t (C-4),

27.02 t (C-5), 19.57 q (Me-9), 18.71 q (Me-10), 13.37 q (Me-8). $[\alpha]_D^{28}$ =-34 ($CHCl_3$, c=0.7). HRMS: calcd for $C_{17}H_{29}O_3N$: 295.2142; found: 295.2138.

(1S,2R,4S)-1,7,7-trimethylbicyclo[2.2.1]heptan-2-yl 3-(piperidin-1-yl)propanoate (**13**). Yield: 74%, pale yellow oil. ^1H NMR (δ, ppm, J/Hz): 0.79 (3H, s, Me-10), 0.83 (3H, s, Me-8), 0.86 (3H, s, Me-9), 0.94 (1H, dd, ^2J=13.7, $J_{2endo,\ 1exo}$=3.5, H-2endo), 1.15-1.30 (2H, m, H-4endo, H-5exo), 1.33-1.43 (2H, m, H-16), 1.49-1.57 (4H, m, 2H-15, 2H-17), 1.63 (1H, dd, $J_{3,2exo}$=$J_{3,\ 4exo}$=4.6, H-3), 1.65-1.76 (1H, m, H-4exo), 1.85-1.94 (1H, ddd, ^2J=12.9, $J_{5endo,\ 4endo}$=9.3, $J_{5endo,\ 4exo}$=4.4, H-5endo), 2.30 (1H, m, H-2exo), 2.31-2.42 (4H, br. s, 2H-14, 2H-18), 2.43-2.53 (2H, m, H-12), 2.57-2.66 (2H, m, H-13), 4.86 (1H, ddd, $J_{1exo,\ 2endo}$=10.0, $J_{1exo,\ 2endo}$=3.5, $J_{1exo,5exo}$=2.2, H-1exo). ^{13}C NMR (δ, ppm): 172.62 s (C-11), 79.28 d (C-1), 54.02 t (C-13), 53.78 t (C-14, C-18), 48.32 s (C-6), 47.32 s (C-7), 44.73 d (C-3), 36.21 t (C-2), 32.33 t (C-12), 27.57 t (C-4), 26.66 t (C-5), 25.52 t (C-15, C-17), 23.87 t (C-16), 19.23 q (Me-9), 18.38 q (Me-10), 13.01 q (Me-8). $[\alpha]_D^{24}$ =-31.5 ($CHCl_3$, c=0.8). HRMS: calcd for $C_{17}H_{29}NO_2$: 293.2349; found: 293.2356.

(1S,2R,4S)-1,7,7-trimethylbicyclo[2.2.1]heptan-2-yl 3-(4-methylpiperidin-1-yl)propanoate (**14**). Yield: 77%, colorless oil. ^1H NMR (δ, ppm, J/Hz): 0.79 (3H, s, Me-10), 0.84 (3H, s, Me-8), 0.87 (3H, s, Me-9), 0.89 (3H, d, J=12.1, Me-17), 0.94 (1H, dd, ^2J=13.7, $J_{2endo,\ 1exo}$=3.5, H-2endo), 1.15-1.37 (5H, m, H-4endo, H-5exo, H-15a, H-16, H-18a), 1.53-1.61 (2H, m, H-15e, H-18e), 1.63 (1H, dd, $J_{3,\ 2exo}$=$J_{3,\ 4exo}$=4.6, H-3), 1.65-1.76 (1H, m, H-4exo), 1.84-1.97 (3H, m, H-5endo, H-14a, H-19a), 2.30 (1H, m, H-2exo), 2.45-2.52 (2H, m, H-12), 2.60-2.68 (2H, m, H-13), 2.79-2.86 (2H, m, H-14e, H-19e), 4.86 (1H, ddd, $J_{1exo,\ 2exo}$=10.0, $J_{1exo,\ 2endo}$=3.5, $J_{1exo,\ 5exo}$=2.2, H-1exo). ^{13}C NMR (δ, ppm): 172.61 s (C-11), 79.28 d (C-1), 53.67 t (C-13), 53.26 and 53.22 t (C-14, C-19), 48.32 s (C-6), 47.32 s (C-7), 44.43 d (C-3), 36.22 t (C-2), 33.89 and 33.87 t (C-15, C-18), 32.49 t (C-12), 30.25 d (C-16), 27.57 t (C-4), 26.67 t (C-5), 21.42 q (Me-17), 19.24 q (Me-9), 18.38 q (Me-10), 13.01 q (Me-8). $[\alpha]_D^{24}$ =-30.6 ($CHCl_3$, c=0.68). HRMS: calcd for $C_{19}H_{33}O_2N$: 307.2506; found: 307.2503.

(1S,2R,4S)-1,7,7-trimethylbicyclo[2.2.1]heptan-2-yl 3-(azepan-1-yl)propanoate (*15*). Yield 47%; pale yellow oil. ^1H NMR (δ, ppm, J/Hz): 0.80 (3H, s, Me-10), 0.84 (3H, s, Me-8), 0.87 (3H, s, Me-9), 0.95 (1H, dd, ^2J=13.7, $J_{2endo,\ 1exo}$=3.3, H-2endo), 1.14-1.30 (2H, m, H-4endo, H-5exo), 1.51-1.76 (12H, m, H-4endo, H-5exo, H-3, H-4exo, H-15, H-16, H-17, H-18), 1.87-1.96 (1H, m, H-5endo), 2.26-2.37 (1H, m, H-2exo), 2.47 (2H, t, J=7.2, H-12), 2.59-2.65 (4H, m, H-14, H-19), 2.83 (2H, t, J=7.2, H-13), 4.85 (1H, m, H-1exo). ^{13}C NMR (δ, ppm): 172.68 s (C-11), 79.33 d (C-1), 54.71 t (C-14, C-19), 53.36 t (C-13), 48.30 s (C-6), 47.33 s (C-7), 44.43 d (C-3), 36.26 t (C-2), 32.84 t (C-12), 27.63 t (C-15, C-18), 27.57 t (C-4), 26.68 t (C-5), 26.48 t (C-16, C-17), 19.25 q (Me-9), 18.39 q (Me-8), 13.03 q (Me-10). HR-MS: 307.2507 (M^+, $C_{19}H_{33}O_2N_1$; calcd 307.2506).

(1S,2R,4S)-1,7,7-trimethylbicyclo[2.2.1]heptan-2-yl 3-(4-methylpiperazin-1-yl)propanoate (**16**). Yield: 91%, pale yellow oil. ^1H NMR (δ, ppm, J/Hz): 0.78 (3H, s, Me-10), 0.82 (3H, s, Me-8), 0.85 (3H, s, Me-9), 0.93 (1H, dd, ^2J=13.7, $J_{2endo,\ 1exo}$=3.5, H-2endo), 1.10-1.30 (2H, m, H-4endo, H-5exo), 1.62 (1H, dd, $J_{3,\ 2exo}$=$J_{3,\ 4exo}$=4.6, H-3), 1.62-1.74 (1H, m, H-4exo), 1.83-1.91 (1H, ddd, ^2J=12.9, $J_{5endo,\ 4endo}$=9.3, $J_{5endo,\ 4exo}$=4.4, H-5endo), 2.22 (3H, s, Me-16), 2.29 (1H, m, H-2exo), 2.43-2.47 (2H, m, H-12), 2.32-2.57 (8H, br. s., 2H-14, 2H-15, 2H-17, 2H-18), 2.62-2.67 (2H, m, H-13), 4.85 (1H, ddd, $J_{1exo,\ 2exo}$=10.0, $J_{1exo,\ 2endo}$=3.5, $J_{1exo,\ 5exo}$=2.2, H-1exo). ^{13}C NMR (δ, ppm): 172.61 s (C-11), 79.64 d (C-1), 54.94 t (C-14, C-18), 53.56 t (C-13), 52.71 (C-15, C-17), 48.62 s (C-6), 47.62 s (C-7), 45.90 k (Me-16), 44.71 d (C-3), 36.50 t (C-2), 32.66 t (C-12), 27.88 t (C-4), 26.97 t (C-5),

19.54 q (Me-9), 18.68 q (Me-10), 13.35 q (Me-8). $[\alpha]_D^{25}$ =-30.6 ($CHCl_3$, c=0.68). HRMS: calcd for $C_{18}H_{32}O_2N_2$: 308.2458; found: 308.2459.

(1S,2R,4S)-1,7,7-Trimethylbicyclo[2.2.1]heptan-2-yl 3-(4-ethylpiperazin-1-yl)propanoate (**17**). Yield: 68%, pale yellow oil. ^{1}H NMR (δ, ppm, J/Hz): 0.79 (3H, s, Me-10), 0.82 (3H, s, Me-8), 0.86 (3H, s, Me-9), 0.94 (1H, dd, ^{2}J=13.7, $J_{2endo,\ 1exo}$=3.5, H-2endo), 1.03 (3H, t, J=7.24, Me-17), 1.13-1.30 (2H, m, H-4endo, H-5exo), 1.63 (1H, dd, $J_{3,\ 2exo}$=$J_{3,\ 4exo}$=4.6, H-3), 1.64-1.75 (1H, m, H-4exo), 1.83-1.93 (1H, ddd, ^{2}J=12.9, $J_{5endo,\ 4endo}$=9.3, $J_{5endo,\ 4exo}$=4.4, H-5endo), 2.30 (1H, m, H-2exo), 2.35 (2H, t, J=7.2, H-16), 2.44-2.50 (2H, m, H-12), 2.32-2.63 (8H, br. s., 2H-14, 2H-15, 2H-18, 2H-19), 2.63-2.69 (2H, m, H-13), 4.86 (1H, ddd, $J_{1exo,2exo}$=10.0, $J_{1exo,2endo}$=3.5, $J_{1exo,5exo}$=2.2, H-1exo). ^{13}C NMR (δ, ppm): 172.63 s (C-11), 79.64 d (C-1), 53.57 t (C-13), 52.72 t (C-14, C-19), 52.61 (C-15, C-18), 52.11 t (C-16), 48.62 s (C-6), 47.61 s (C-7), 44.71 d (C-3), 36.50 t (C-2), 32.65 t (C-12), 27.87 t (C-4), 26.96 t (C-5), 19.53 k (Me-9), 18.66 q (Me-10). 13.32 q (Me-8), 11.81 q (Me-17). $[\alpha]_D^{25}$ =-25.5 ($CHCl_3$, c=0.94). HRMS: calcd for $C_{19}H_{34}O_2N_2$: 322.2615; found: 322.2612.

*(1S,2R,4S)-1,7,7-trimethylbicyclo[2.2.1]heptan-2-yl 3-(4-benzylpiperazin-1-yl)propanoate (**18**).* Yield 45%; pale yellow oil. ^{1}H NMR (δ, ppm, J/Hz): 0.80 (3H, s, Me-10), 0.84 (3H, s, Me-8), 0.88 (3H, s, Me-9), 0.95 (1H, dd, ^{2}J=13.7, $J_{2endo,\ 1exo}$=3.3, H-2endo), 1.15-1.31 (2H, m, H-4endo, H-5exo), 1.62-1.67 (1H,m, H-3), 1.66-1.76 (1H, m, H-4exo), 1.85-1.94 (1H, m, H-5endo), 2.26-2.36 (1H, m, H-2exo), 2.37-2.54 (10H, br. s., H-12, H-14, H-15, H-16), 2.65-2.69 (2H, m, H-13), 3.48 (2H, s, H-18), 4.86 (1H, m, H-1exo), 7.19-7.23 (1H, m, H-22), 7.26-7.30 (4H, m, H-20, H-21, H-23, H-24). ^{13}C NMR (δ, ppm): 172.74 s (C-11), 137.96 s (C-19), 129.08 d (C-20, C-24), 128.07 d (C-21, C-23), 126.89 d (C-22), 79.70 d (C-1), 62.92 t (C-18), 53.60 t (C-13), 52.91 and 52.75 t (C-14, C-15, C-16, C-17), 48.66 s (C-6), 47.66 s (C-7), 44.73 d (C-3), 36.54 t (C-2), 32.69 t (C-12), 27.91 t (C-4), 26.99 t (C-5), 19.58 q (Me-9), 18.71 q (Me-8), 13.38 q (Me-10). HR-MS: 384.2767 (M^+, $C_{24}H_{36}O_2N_2$; calcd 384.2771).

(1S,2R,4S)-1,7,7-Trimethylbicyclo[2.2.1]heptan-2-yl 3-(dimethylamino)propanoate (***19***). A solution of (1S,2R,4S)-1,7,7-trimethylbicyclo[2.2.1]heptan-2-yl 3-chloropropanoate (2.8 mmol) and CH_3CN (7 mL) was treated with an excess of dimethylamine (1.5 mL 40% wt.) solution in ethanol and K_2CO_3 (7 mmol) was added. The mixture was refluxed for 12, after which the precipitate was filtered off and the solvent was removed at reduced pressure. Then, CH_2Cl_2 was added to the resulting mixture, after which the solution was diluted with brine and extracted twice with CH_2Cl_2. The combined organic layer was dried with anhydrous Na_2SO_4 and evaporated. The crude residues were purified via silica gel column chromatography (hexane-ethylacetate eluent). Yield 53%; pale yellow oil. ^{1}H NMR (δ, ppm, J/Hz): 0.78 (3H, s, Me-10), 0.82 (3H, s, Me-8), 0.86 (3H, s, Me-9), 0.93 (1H, dd, ^{2}J=13.7, $J_{2endo,\ 1exo}$=3.3, H-2endo), 1.14-1.30 (2H, m, H-4endo, H-5exo), 1.61-1.75 (2H, m, H-3, H-4exo), 1.85-1.94 (1H, m, H-5endo), 2.21 (6H, s, M-14, Me-15), 2.25-2.35 (1H, m, H-2exo), 2.42-2.61 (4H, AB, J=7.1, H-12, H-13), 4.85 (1H, m, H-1exo). ^{13}C NMR (δ, ppm): 172.63 s (C-11), 79.75 d (C-1), 54.78 t (C-13), 48.65 s (C-6), 47.66 s (C-7), 45.05 q (Me-14, Me-15), 44.82 d (C-3), 36.61 t (C-2), 33.17 t (C-12), 27.90 t (C-4), 27.03 t (C-5), 19.56 q (Me-9), 18.69 q (Me-8), 13.29 q (Me-10). HR-MS: 253.2033 (M^+, $C_{15}H_{27}O_2N$; calcd 253.2036).

(1S,2R,4S)-1,7,7-Trimethylbicyclo[2.2.1]heptan-2-yl 3-(diethylamino)propanoate (***20***). A solution of (1S,2R,4S)-1,7,7-trimethylbicyclo[2.2.1]heptan-2-yl 3-chloropropanoate (2.8 mmol) and CH_3CN (7 mL) was treated with an excess of diethylamine and K_2CO_3 (7 mmol) was

added. The mixture was refluxed for 12, after which the precipitate was filtered off and the solvent was removed at reduced pressure. Then, CH_2Cl_2 was added to the resulting mixture, after which the solution was diluted with brine and extracted twice with CH_2Cl_2. The combined organic layer was dried with anhydrous Na_2SO_4 and evaporated. The crude residues were purified via silica gel column chromatography (hexane-ethylacetate eluent). Yield 58%; pale yellow oil. ^{1}H NMR (δ, ppm, J/Hz): 0.79 (3H, s, Me-10), 0.83 (3H, s, Me-8), 0.86 (3H, s, Me-9), 0.93 (1H, dd, ^{2}J=13.7, $J_{2endo, 1exo}$=3.3, H-2endo), 0.99 (6H, t, J=7.2, Me-16, Me-17), 1.15-1.29 (2H, m, H-4endo, H-5exo), 1.61-1.64 (1H, m, H-3), 1.65-1.73 (1H, m, H-4exo), 1.86-1.92 (1H, m, H-5endo), 2.21 (6H, s, M-14, Me-15), 2.26-2.34 (1H, m, H-2exo), 2.39-2.44 (2H, m, H-12), 2.48 (4H, q, J=7.2, H-14, H-15), 2.74-2.80 (2H, m, H-13), 4.84 (1H, m, H-1exo). ^{13}C NMR (δ, ppm): 173.08 s (C-11), 79.59 d (C-1), 48.57 s (C-6), 48.23 t (C-13), 47.61 s (C-7), 46.51 t (C-14, C-15), 44.71 d (C-3), 36.56 t (C-2), 32.35 t (C-12), 27.86 t (C-4), 26.96 t (C-5), 19.54 q (Me-9), 18.68 q (Me-8), 13.33 q (Me-10), 11.70 q (Me-16, Me-17). HR-MS: 281.2348 (M^+, $C_{17}H_{31}O_2N_1$; calcd 281.2349).

(1S,2R,4S)-1,7,7-Trimethylbicyclo[2.2.1]heptan-2-yl 3-(dibutylamino)propanoate (**21**). The synthesis was carried out according to the general procedure. Yield: 69%, yellow oil. ^{1}H NMR (δ, ppm, J/Hz): 0.79 (3H, s, Me-9), 0.83 (3H, s, Me-10), 0.86 (3H, s, Me-8), 0.87 (6H, t, J=7.2, Me-20, Me-21), 0.93 (1H, m, H-2endo), 1.14-1.43 (10H, m, H-4endo, H-5exo, 2H-16, 2H-17, 2H-18, 2H-19), 1.63 (1H, m, H-3), 1.65-1.75 (1H, m, H-4exo), 1.86-1.95 (1H, m, H-5endo), 2.25-2.34 (1H, m, H-2exo), 2.32-2.44 (6H, m, 2H-12, 2H-14, 2H-15), 2.72-2.79 (2H, m, H-13), 4.84 (1H, ddd, $J_{1exo, 2exo}$=10.0, $J_{1exo, 2endo}$=3.5, $J_{1exo, 5exo}$=2.2, H-1exo). ^{13}C NMR (δ, ppm): 173.18 s (C-11), 79.57 d (C-1), 53.45 t (C-14, S-15), 49.48 t (C-13), 48.57 s (C-6), 47.63 s (C-7), 44.74 d (C-3), 36.59 t (C-2), 32.48 t (C-12), 29.23 t (C-16, C-17), 27.88 t (C-4), 26.98 t (C-5), 20.53 t (C-18, C-19), 19.56 q (Me-9), 18.69 q (Me-10), 13.94 q (Me-20, Me-21), 13.35 q (Me-8). $[\alpha]_D^{25}$=-28.8 ($CHCl_3$, c=0.82). HRMS: calcd for $C_{21}H_{39}O_2N$: 337.2975; found: 337.2970.

General synthetic procedure for compounds 22-28

A mixture of 1,7,7-trimethylbicyclo[2.2.1]heptan-2-yl 4-chlorobutanoate (2 mmol), the corresponding amine (2.2 mmol), Et_3N (2.2 mmol) and 15 ml CH_2Cl_2 were refluxed for 12 h. After completion, the mixture was concentrated in a vacuum. Brine and $CHCl_3$ were added to the residue, and then the mixture was extracted twice with $CHCl_3$. The combined organic phase was dried over Na_2SO_4 and the solvent was removed in vacuo. The crude product was purified by silica gel CC (eluent: hexane–ethyl acetate).

(1S,2R,4S)-1,7,7-Trimethylbicyclo[2.2.1]heptan-2-yl 4-(piperidin-1-yl)butanoate (**22**). Yield: 71%, yellow oil. ^{1}H NMR (δ, ppm, J/Hz): 0.79 (3H, s, Me-10), 0.84 (3H, s, Me-8), 0.87 (3H, s, Me-9), 0.92 (1H, dd, ^{2}J=13.7, $J_{2endo, 1exo}$=3.5, H-2endo), 1.14-1.31 (2H, m, H-4endo, H-5exo), 1.35-1.44 (2H, m, H-17), 1.48-1.59 (4H, m, 2H-16, 2H-18), 1.61-1.65 (1H, m, H-3), 1.65-1.74 (1H, m, H-4exo), 1.79 (2H, p, J=7.4, H-13), 1.85-1.94 (1H, m, H-5endo), 2.24-2.36 (9H, m, H-2exo, 2H-12, 2H-14, H-15, H-19), 4.85 (1H, ddd, $J_{1exo, 2endo}$=10.0, $J_{1exo, 2endo}$=3.5, $J_{1exo,5exo}$=2.2, H-1exo). ^{13}C NMR (δ, ppm): 173.68 s (C-11), 79.51 d (C-1), 58.34 t (C-14), 54.34 t (C-15, C-19), 48.52 s (C-6), 47.57 s (C-7), 44.67 d (C-3), 36.60 t (C-2), 32.56 t (C-12), 27.83 t (C-4), 26.90 t (C-5), 25.70 t (C-16, C-18), 24.19 t (C-17), 22.12 t (C-13), 19.49 q (Me-9), 18.63 q (Me-10), 13.32 q (Me-8). $[\alpha]_D^{30}$=-38.6 ($CHCl_3$, c=0.8). HRMS: calcd for $C_{19}H_{33}O_2N_1$: 307.2506; found: 307.2504.

(1S,2R,4S)-1,7,7-Trimethylbicyclo[2.2.1]heptan-2-yl 4-(4-methylpiperidin-1-yl)butanoate (**23**). Yield: 57%, yellow oil. ^{1}H NMR (δ, ppm, J/Hz): 0.80 (3H, s, Me-10), 0.84 (3H, s, Me-8), 0.87 (3H, s, Me-9), 0.88 (3H, d, J=12.1, Me-20), 0.93 (1H, dd, ^{2}J=13.7, $J_{2endo, 1exo}$=3.5, H-2endo), 1.15-1.33 (5H, m, H-4endo, H-5exo, H-17a, H-19, H-18a), 1.54-1.60 (2H, m, H-17e, H-18e), 1.62-1.66 (1H, m, H-3), 1.65-1.90 (6H, m, H-4exo, H-5endo, H-15a, H-16a, H-13), 2.25-2.36 (7H, m, H-2exo, H-12, H-13), 2.81-2.87 (2H, m , H-15e, H-16e), 4.79-4.88 (1H, m, H-1exo). ^{13}C NMR (δ, ppm): 173.69 s (C-11), 79.64 d (C-1), 57.96 t (C-14), 53.81 t (C-15, C-16), 48.62 s (C-6), 47.66 s (C-7), 44.76 d (C-3), 36.70 t (C-2), 33.92 t (C-17, C-18), 32.57 t (C-12), 30.58 d (C-19), 27.92 t (C-4), 27.00 t (C-5), 22.17 t (C-13), 21.71 q (Me-20), 19.58 q (Me-9), 18.71 q (Me-10), 13.41 q (Me-8). $[\alpha]_D^{25}$ =-35.7 (CHCl$_3$, c=0.6). HRMS: calcd for $C_{20}H_{35}O_2N_1$: 321.2662; found: 321.2660.

(1S,2R,4S)-1,7,7-Trimethylbicyclo[2.2.1]heptan-2-yl 4-(4-methylpiperazin-1-yl)butanoate (**24**). Yield: 56%, pale yellow oil. ^{1}H NMR (δ, ppm, J/Hz): 0.77 (3H, s, Me-10), 0.82 (3H, s, Me-8), 0.85 (3H, s, Me-9), 0.90 (1H, dd, ^{2}J=13.7, $J_{2endo, 1exo}$=3.5, H-2endo), 1.13-1.28 (2H, m, H-4endo, H-5exo), 1.62 (1H, dd, $J_{3, 2exo}$=$J_{3, 4exo}$=4.6, H-3), 1.64-1.73 (1H, m, H-4exo), 1.77 (2H, p, J=7.4, H-13), 1.84-1.92 (1H, m, H-5endo), 2.23 (3H, s, Me-19), 2.25-2.56 (12H, m, H-2exo, 2H-12, 2H-14, 2H-15, 2H-16, 2H-17, 2H-18), 4.83 (1H, ddd, $J_{1exo, 2exo}$=10.0, $J_{1exo, 2endo}$=3.5, $J_{1exo, 5exo}$=2.2, H-1exo). ^{13}C NMR (δ, ppm): 173.67 s (C-11), 79.59 d (C-1), 57.55 t (C-14), 54.96 t (C-16, C-18), 52.98 t (C-15, C-17), 48.59 s (C-6), 47.63 s (C-7), 45.91 q (Me-19), 44.71 d (C-3), 36.68 t (C-2), 32.47 t (C-12), 27.90 t (C-4), 26.97 t (C-5), 22.15 t (C-13), 19.56 q (Me-9), 18.69 q (Me-10), 13.41 q (Me-8). $[\alpha]_D^{29}$ =-30.36 (CHCl$_3$, c=1.1). HRMS: calcd for $C_{19}H_{34}O_2N_2$: 322.2615; found: 322.2622.

(1S,2R,4S)-1,7,7-Trimethylbicyclo[2.2.1]heptan-2-yl 4-(4-(2-hydroxyethyl)piperazin-1-yl)butanoate (**25**). Yield 27%; pale yellow oil. ^{1}H NMR (δ, ppm, J/Hz): 0.77 (3H, s, Me-10), 0.82 (3H, s, Me-8), 0.85 (3H, s, Me-9), 0.90 (1H, dd, ^{2}J=14.2, $J_{2endo, 1exo}$=3.2, H-2endo), 1.10-1.30 (2H, m, H-4endo, H-5exo), 1.59-1.64 (1H, m, H-3), 1.66-1.93 (4H, m, H-4exo, H-13, H-5endo), 2.27-2.36 (5H, m, H-2exo, H-12, H-14), 2.36-2.55 (10H, br. s., H-15, H-16, H-17, H-18, H-19), 2.82 (1H, br. s., OH), 3.56 (2H, t, J=5.4, H-20), 4.83 (1H, m, H-1exo). ^{13}C NMR (δ, ppm): 173.58 s (C-11), 79.57 d (C-1), 59.18 t (C-20), 57.59 t (C-14), 57.46 t (C-19), 52.96 and 52.71 t (C-15, C-16, C-17, C-18), 48.57 s (C-6), 47.60 s (C-7), 44.71 d (C-3), 36.66 t (C-2), 32.38 t (C-12), 27.88 t (C-4), 26.95 t (C-5), 22.10 t (C-13), 19.52 q (Me-9), 18.65 q (Me-8), 13.36 q (Me-10). HR-MS: 352.2716 (M^+, $C_{20}H_{36}O_3N_2$; calcd 352.2721).

(1S,2R,4S)-1,7,7-trimethylbicyclo[2.2.1]heptan-2-yl 4-(4-(2-aminoethyl)piperazin-1-yl)butanoate (**26**). Yield 35%; brown oil. ^{1}H NMR (δ, ppm, J/Hz): 0.78 (3H, s, Me-10), 0.83 (3H, s, Me-8), 0.86 (3H, s, Me-9), 0.91 (1H, dd, ^{2}J=14.2, $J_{2endo, 1exo}$=3.2, H-2endo), 1.13-1.30 (2H, m, H-4endo, H-5exo), 1.61-1.66 (1H, m, H-3), 1.66-1.94 (4H, m, H-4exo, H-13, H-5endo), 1.96-2.04 (2H, br. s., NH_2), 2.26-2.56 (15H, m, H-2exo, H-12, H-14, H-15, H-16, H-17, H-18, H-19), 4.84 (1H, m, H-1exo). ^{13}C NMR (δ, ppm): 173.67 s (C-11), 79.66 d (C-1), 57.48 t (C-14), 56.12 t (C-19), 52.88 and 52.62 t (C-15, C-16, C-17, C-18), 48.60 s (C-6), 47.65 s (C-7), 44.71 d (C-3), 36.69 t (C-2), 34.29 t (C-20), 32.39 t (C-12), 27.91 t (C-4), 26.97 t (C-5), 22.08 t (C-13), 19.56 q (Me-9), 18.69 q (Me-8), 13.42 q (Me-10). Anal. Calcd for $C_{20}H_{37}O_2N_3$, C, 68.33; H, 10.61; N, 11.95. Found, %: C, 68.63; H, 10.07; N, 11.53.

Ethyl 4-(4-oxo-4-((1S,2R,4S)-1,7,7-trimethylbicyclo[2.2.1]heptan-2-yloxy)butyl)piperazine-1-carboxylate (**27**). Yield 37%; pale yellow oil. ^{1}H NMR (δ, ppm, J/Hz): 0.79 (3H, s, Me-10), 0.83 (3H, s, Me-8), 0.87 (3H, s, Me-9), 0.91 (1H, dd, ^{2}J=14.2, $J_{2endo, 1exo}$=3.2, H-2endo), 1.14-1.30 (5H,

m, H-4endo, H-5exo, Me-21), 1.61-1.65 (1H, m, H-3), 1.65-1.74 (1H, m, H-4exo), 1.78 (2H, quint., J = 7.2, H-13), 1.84-1.92 (1H, m, H-5endo), 2.25-2.42 (9H, m, H-2exo, H-12, H-14, H-15, H-16), 3.39-3.47 (4H, br. s., H-17, H-18), 4.09 (2H, q, J=7.05, H-20), 4.85 (1H, m, H-1exo). ^{13}C NMR (δ, ppm): 173.48 s (C-11), 155.31 s (C-19), 79.78 d (C-1), 61.26 t (C-20), 57.44 t (C-14), 52.64 t (C-15, C-16), 48.64 s (C-6), 47.68 s (C-7), 44.79 d (C-3), 43.25 t (C-17, C-18), 36.72 t (C-2), 32.19 t (C-12), 27.94 t (C-4), 27.02 t (C-5), 21.73 t (C-13), 19.57 q (Me-9), 18.70 q (Me-8), 14.53 q (Me-21), 13.41 q (Me-10). HR-MS: 380.2666 (M^+, $C_{21}H_{36}O_4N_2$; calcd 380.2670).

(1S,2R,4S)-1,7,7-trimethylbicyclo[2.2.1]heptan-2-yl 4-(azepan-1-yl)butanoate (**28**). Yield 45%; yellow oil. ^1H NMR (δ, ppm, J/Hz): 0.79 (3H, s, Me-10), 0.84 (3H, s, Me-8), 0.87 (3H, s, Me-9), 0.92 (1H, dd, ^2J=13.7, $J_{2endo,\ 1exo}$=3.3, H-2endo), 1.15-1.30 (2H, m, H-4endo, H-5exo), 1.51-1.66 (11H, m, H-4endo, H-5exo, H-3, H-16, H-17, H-18, H-19), 1.66-1.81 (3H, m, H-4exo, H-13), 1.87-1.95 (1H, m, H-5endo), 2.26-2.37 (3H, m, H-2exo, H-12), 2.48 (2H, t, J=7.4, H-14), 2.58-2.64 (4H, m, H-15, H-20), 4.85 (1H, m, H-1exo). ^{13}C NMR (δ, ppm): 173.22 s (C-11), 80.01 d (C-1), 56.71 t (C-14), 54.71 t (C-15, C-20), 48.61 s (C-6), 47.67 s (C-7), 44.73 d (C-3), 36.65 t (C-2), 31.69 t (C-12), 27.88 t (C-4), 26.95 t (C-5), 26.84 t (C-16, C-19), 25.69 t (C-17, C-18), 21.14 t (C-13), 19.54 q (Me-9), 18.67 q (Me-8), 13.39 q (Me-10). HR-MS: 321.2659 (M^+, $C_{20}H_{35}O_2N_1$; calcd 321.2662).

(1S,2R,4S)-1,7,7-trimethylbicyclo[2.2.1]heptan-2-yl 4-(dimethylamino)butanoate (**29**). A solution of 1,7,7-trimethylbicyclo[2.2.1]heptan-2-yl 4-chlorobutanoate (2.8 mmol) and CH_3CN (7 mL) was treated with an excess of dimethylamine (1.5 mL 40% wt.) solution in ethanol and K_2CO_3 (7 mmol) was added. The mixture was refluxed for 12, after which the precipitate was filtered off and the solvent was removed at reduced pressure. Then, CH_2Cl_2 was added to the resulting mixture, after which the solution was diluted with brine and extracted twice with CH_2Cl_2. The combined organic layer was dried with anhydrous Na_2SO_4 and evaporated. The crude residues were purified via silica gel column chromatography (hexane-ethylacetate eluent). Yield 47%; pale yellow oil. ^1H NMR (δ, ppm, J/Hz): 0.79 (3H, s, Me-10), 0.83 (3H, s, Me-8), 0.86 (3H, s, Me-9), 0.91 (1H, dd, ^2J=13.7, $J_{2endo,\ 1exo}$=3.3, H-2endo), 1.14-1.30 (2H, m, H-4endo, H-5exo), 1.60-1.75 (2H, m, H-3, H-4exo), 1.77-1.93 (3H, m, H-5endo, H-13), 2.28 (6H, s, M-15, Me-16), 2.29-2.40 (5H, m, H-2exo, H-12, H-14), 4.84 (1H, m, H-1exo). ^{13}C NMR (δ, ppm): 173.68 s (C-11), 79.59 d (C-1), 58.70 t (C-14), 48.55 s (C-6), 47.61 s (C-7), 45.20 q (Me-15, Me-16), 44.69 d (C-3), 36.63 t (C-2), 32.26 t (C-12), 27.87 t (C-4), 26.92 t (C-5), 22.82 t (C-13), 19.54 q (Me-9), 18.67 q (Me-8), 13.36 q (Me-10). HR-MS: 267.1197 (M^+, $C_{16}H_{29}NO_2$; calcd 267.2193).

Synthesis of isobornilamine-based amides.

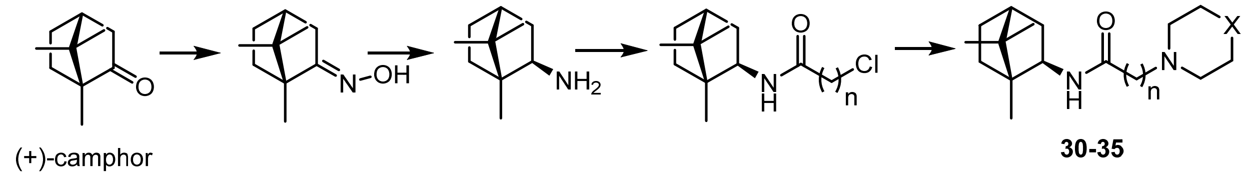

30-35

2-Chloro-N-((1R,2R,4R)-1,7,7-trimethylbicyclo[2.2.1]heptan-2-yl)acetamide. To the mixture of isobornilamine (0.012 mol) which has been synthesized by a procedure described previously[1], and Et_3N (0.012 mol) in 20 ml dry CH_2Cl_2 at 15–18 °C in an Ar atmosphere was added chloroacetyl chloride (0.017 mol), and the mixture was stirred at room temperature for 24 h. The organic layers were washed with brine and extracted with $CHCl_3$. The combined

organic phase was dried over anhydrous Na_2SO_4 and the solvent was removed. The resulting residue was used in next step without any further purification.

3-Chloro-N-((1R,2R,4R)-1,7,7-trimethylbicyclo[2.2.1]heptan-2-yl)propanamide. To the solution of 3-chloropropanoic acid in dry CH_2Cl_2, excess oxalyl chloride and N,N-dimethylformamide (one drop) were added. The mixture was stirred at room temperature for 4 h in an Ar atmosphere. The excess oxalyl chloride was removed on a rotary evaporator. The resulting 3-chloropropanoyl chloride was used in a further reaction immediately. To the solution of amine 1 (15 mmol) in CH_2Cl_2 was added the 3-chloropropanoyl chloride (20 mmol) in CH_2Cl_2 (5 ml) and Et_3N (15 mmol) at 0–5 °C, and the mixture was stirred at room temperature for 24 h in an atmosphere of Ar. The organic layer was washed with brine and extracted with CH_2Cl_2. The combined organic phase was dried over anhydrous Na_2SO_4 and the solvent was removed. The resulting residue was used in next step without any further purification.

General synthetic procedure for compounds 30–35

A mixture of acetamide (2 mmol), the corresponding amine (2.2 mmol), Et_3N (2.2 mmol) and 10 ml CH_2Cl_2 was refluxed for 12 h. After completion, the mixture was washed brine and extracted twice with $CHCl_3$. The combined organic phase was dried over Na_2SO_4 and the solvent was removed in vacuo. The crude product was purified by silica gel CC (eluent: hexane–ethyl acetate).

2-(4-Methylpiperidin-1-yl)-N-((1R,2R,4R)-1,7,7-trimethylbicyclo[2.2.1]heptan-2-yl)acetamide (**30**). Yield: 45%, yellow oil. ^1H NMR (δ, ppm, J/Hz): 0.79 (3H, s, Me-10), 0.82 (3H, s, Me-8), 0.89 (3H, d, J=6.3, Me-18), 0.91 (3H, s, Me-9), 1.08-1.4 (5H, m, H-4endo, H-5endo, H-14a, H-15, H-16a), 1.49-1.67 (4H, m, H-5exo, H-4exo, H-14e, H-16e), 1.70-1.74 (1H, m, H-3), 1.82 (1H, dd, J=9.0, 14.0, H-2exo), 1.85-1.94 (2H, m, H-13e, H-17e), 2.68-2.80 (2H, m, H-13e, H-17e), 2.90 (2H, AB-d, H-12), 3.81-3.87 (1H, m, H-1exo), 7.49 (1H, N-H). ^{13}C NMR (δ, ppm): 169.56 s (C-11), 61.61 t (C-12), 55.57 d (C-1), 54.24 t (C-13, C-17), 48.24 s (C-6), 46.89 s (C-7), 44.71 d (C-3), 39.06 t (C-2), 35.70 t (C-5), 34.60 t (C-14, C-16), 29.88 d (C-15), 26.88 t (C-4), 21.67 q (Me-18), 20.08 q, 19.90 q (Me-8, Me-9), 11.84 q (Me-10). $[\alpha]_D^{24}$ = -27.6 ($CHCl_3$, c=1.1). HRMS: calcd for $C_{18}H_{32}O_1N_2$: 292.2509 found: 292.2514.

2-(4-Methylpiperazin-1-yl)-N-((1R,2R,4R)-1,7,7-trimethylbicyclo[2.2.1]heptan-2-yl)acetamide (**31**). Yield: 63%, yellow oil. ^1H NMR (δ, ppm, J/Hz): 0.75 (3H, s, Me-10), 0.78 (3H, s, Me-8), 0.86 (3H, s, Me-9), 1.05-1.12 (1H, m, H-4endo), 1.19-1.27 (1H, m, H-5endo), 1.45-1.54 (2H, m, H-5exo, H-2endo), 1.59-1.66 (1H, m, H-4exo), 1.66-1.70 (1H, m, H-3), 1.78 (1H, dd, J=9.0, 14.0, H-2exo), 2.21 (3H, s, Me-17), 2.29-2.55 (8H, m, H-13, H-14, H-15, H-16), 2.91 (2H, AB-d, J=3.8, H-12), 3.80 (1H, dt, J=4.9, 9.2, H-1exo), 7.33 (1H, N-H). ^{13}C NMR (δ, ppm): 168.89 s (C-11), 60.99 t (C-12), 55.58 d (C-1), 55.16 t (C-13, C-14), 53.19 t (C-16, C-15), 48.18 s (C-6), 46.86 s (C-7), 45.74 q (Me-17), 44.64 d (C-3), 39.01 t (C-2), 35.62 t (C-5), 26.80 t (C-4), 20.00 q, 19.98 q (Me-8, Me-9), 11.83 q (Me-10). $[\alpha]_D^{24}$ = -26.7 ($CHCl_3$, c=0.9). HRMS: calcd for $C_{17}H_{31}O_1N_3$: 293.2462; found: 293.2460.

2-Morpholino-N-((1R,2R,4R)-1,7,7-trimethylbicyclo[2.2.1]heptan-2-yl)acetamide (**32**). Yield: 59%, pale yellow oil. ^1H NMR (δ, ppm, J/Hz): 0.78 (3H, s, Me-10), 0.82 (3H, s, Me-8), 0.90 (3H,

s, Me-9), 1.09-1.17 (1H, m, H-4endo), 1.23-1.31 (1H, m, H-5endo), 1.48-1.58 (2H, m, H-5exo, H-2endo), 1.63-1.70 (1H, m, H-4exo), 1.73 (1H, t, $J_{3,2exo}$= $J_{3,4exo}$ =3.8, H-3), 1.83 (1H, dd, J=9.0, 14.0, H-2exo), 2.49-2.55 (4H, m, H-13, H-14), 2.95 (2H, AB-d, J=1.5, H-12), 3.66-3.74 (4H, m, H-15, H-16), 3.85 (1H, dt, J=4.9, 9.2, H-1exo), 7.33 (1H, N-H).^{13}C NMR (δ, ppm): 168.6 s (C-11), 67.02 t (C-15, C-16), 61.65 t (C-12), 55.74 d (C-1), 53.73 t (C-13, C-14), 48.30 s (C-6), 46.95 s (C-7), 44.75 d (C-3), 39.12 t (C-2), 35.71 t (C-5), 26.88 t (C-4), 20.08 q, 19.96 q (Me-8, Me-9), 11.93 q (Me-10). $[\alpha]_D^{24}$ = -29.8 ($CHCl_3$, c=1.1). HRMS: calcd for $C_{16}H_{28}O_2N_2$: 280.2145; found: 280.2139.

3-Morpholino-N-((1R,2R,4R)-1,7,7-trimethylbicyclo[2.2.1]heptan-2-yl)propanamide (**33**). Yield: 43%, pale yellow oil. ^{1}H NMR (δ, ppm, J/Hz): 0.80 (3H, s, Me-10), 0.81 (3H, s, Me-8), 0.91 (3H, s, Me-9), 1.07-1.15 (1H, m, H-4endo), 1.20-1.29 (1H, m, H-5endo), 1.45-1.56 (2H, m, H-5exo, H-2endo), 1.61-1.71 (2H, m, H-4exo, H-3), 1.82 (1H, dd, J=9.0, 14.0, H-2exo), 2.33-2.37 (2H, m, H-12), 2.40-2.47 (4H, br. s., H-14, H-15), 2.50-2.57 (2H, m, H-13), 3.63-3.73 (4H, m, H-16, H-17), 3.84 (1H, dt, J=4.9, 9.2, H-1exo), 8.10 (1H, N-H).^{13}C NMR (δ, ppm): 171.2 s (C-11), 66.37 t (C-16, C-17), 55.96 d (C-1), 54.78 t (C-13), 52.93 t (C-14, C-15), 48.10 s (C-6), 46.86 s (C-7), 44.68 d (C-3), 39.16 t (C-2), 35.81 t (C-5), 31.27 t (C-12), 26.83 t (C-4), 20.56 q, 20.03 q (Me-8, Me-9), 11.81 q (Me-10). $[\alpha]_D^{26}$= 27.4 ($CHCl_3$, c=0.6). HRMS: calcd for $C_{17}H_{30}O_2N_2$: 294.2302 found: 294.2300.

3-(4-Methylpiperazin-1-yl)-N-((1R,2R,4R)-1,7,7-trimethylbicyclo[2.2.1]heptan-2-yl)propanamide (**34**). Yield: 53%, pale yellow oil. ^{1}H NMR (δ, ppm, J/Hz): 0.80 (3H, s, Me-8), 0.81 (3H, s, Me-10), 0.91 (3H, s, Me-9), 1.08-1.17 (1H, m, H-4endo), 1.21-1.30 (1H, m, H-5endo), 1.45-1.56 (2H, m, H-5exo, H-2endo), 1.61-1.71 (2H, m, H-4exo, H-3), 1.82 (1H, dd, J=9.0, 14.0, H-2exo), 2.26 (3H, s, Me-18), 2.27-2.69 (8H, br.s., H-14, H-15, H-16, H-17), 2.30-2.40 (2H, m, H-12), 2.48-2.60 (2H, m, H-13), 3.84 (1H, dt, J=4.9, 9.2, H-1exo), 8.24 (1H, N-H).^{13}C NMR (δ, ppm): 171.58 s (C-11), 56.02 d (C-1), 54.58 t (C-13), 54.33 t (C-14, C-15), 52.50 t (C-16, C-17), 48.21 s (C-6), 46.93 s (C-7), 45.87 q (Me-18), 44.76 d (C-3), 39.14 t (C-2), 35.88 t (C-5), 31.63 t (C-12), 26.92 t (C-4), 20.92 q, 20.13 q (Me-8, Me-9), 11.89 q (Me-10). $[\alpha]_D^{25}$=18.5 (CHCl3, c=0.7). HRMS: calcd for $C_{18}H_{33}O_1N_3$: 307.2618; found: 307.2619.

3-(4-Ethylpiperazin-1-yl)-N-((1R,2R,4R)-1,7,7-trimethylbicyclo[2.2.1]heptan-2-yl)propanamide (**35**). Yield: 62%, yellow oil. ^{1}H NMR (δ, ppm, J/Hz): 0.80 (3H, s, Me-10), 0.81 (3H, s, Me-8), 0.91 (3H, s, Me-9), 1.05 (3H, t, J=7.3, Me-19), 1.08-1.14 (1H, m, H-4endo), 1.21-1.29 (1H, m, H-5endo), 1.43-1.54 (2H, m, H-5exo, H-2endo), 1.59-1.69 (2H, m, H-4exo, H-3), 1.79 (1H, dd, J=9.0, 14.0, H-2exo), 2.26-2.75 (14H, m, H-12, H-13, H-14, H-15, H-16, H-17, H-18), 3.82 (1H, dt, J=4.9, 9.2, H-1exo), 8.22 (1H, N-H).^{13}C NMR (δ, ppm): 171.59 s (C-11), 56.00 d (C-1), 54.31 t (C-13), 52.44 t (C-14, C-15), 52.22 t (C-16, C-17), 48.18 s (C-6), 46.90 s (C-7), 44.73 d (C-3), 39.12 t (C-2), 35.87 t (C-5), 31.59 t (C-18), 26.90 t (C-4), 20.90 q, 20.10 q (Me-8, Me-9), 11.87 q (Me-10), 11.77 q (Me-19). $[\alpha]_D^{25}$=-31.3 ($CHCl_3$, c=0.6). HRMS: calcd for $C_{19}H_{35}O_1N_3$: 321.2775; found: 321.2777.

1. Sokolova, A.; Pavlova, A.; Komarova, N.; Ardashov, O.; Shernyukov, A.; Gatilov, Y.; Yarovaya, O.; Tolstikova, T.; Salakhutdinov, N. Synthesis and analgesic activity of new α-truxillic acid derivatives with monoterpenoid fragments. *Med. Chem. Res.* **2016**, *25*, doi:10.1007/s00044-016-1593-z.
